# Structurally-Constrained Relationships between Cognitive States in the Human Brain

**DOI:** 10.1371/journal.pcbi.1003591

**Published:** 2014-05-15

**Authors:** Ann M. Hermundstad, Kevin S. Brown, Danielle S. Bassett, Elissa M. Aminoff, Amy Frithsen, Arianne Johnson, Christine M. Tipper, Michael B. Miller, Scott T. Grafton, Jean M. Carlson

**Affiliations:** 1 Department of Physics, University of California, Santa Barbara, Santa Barbara, California, United States of America; 2 Department of Physics and Astronomy, University of Pennsylvania, Philadelphia, Pennsylvania, United States of America; 3 Department of Biomedical Engineering, University of Connecticut, Storrs, Conneticutt, United States of America; 4 Department of Physics, University of Connecticut, Storrs, Conneticutt, United States of America; 5 Department of Chemical and Biomolecular Engineering, University of Connecticut, Storrs, Conneticutt, United States of America; 6 Department of Marine Sciences, University of Connecticut, Groton, Conneticutt, United States of America; 7 Sage Center for the Study of the Mind, University of California, Santa Barbara, Santa Barbara, California, United States of America; 8 Department of Bioengineering, University of Pennsylvania, Philadelphia, Pennsylvania, United States of America; 9 Department of Electrical and Systems Engineering, University of Pennsylvania, Philadelphia, Pennsylvania, United States of America; 10 Center for the Neural Basis of Cognition, Carnegie Mellon University, Pittsburgh, Pennsylvania, United States of America; 11 Department of Psychological and Brain Sciences, University of California, Santa Barbara, Santa Barbara, California, United States of America; 12 Department of Psychiatry, Faculty of Medicine, University of British Columbia, Vancouver, British Columbia, Canada; Hamburg University, Germany

## Abstract

The anatomical connectivity of the human brain supports diverse patterns of correlated neural activity that are thought to underlie cognitive function. In a manner sensitive to underlying structural brain architecture, we examine the extent to which such patterns of correlated activity systematically vary across cognitive states. Anatomical white matter connectivity is compared with functional correlations in neural activity measured via blood oxygen level dependent (BOLD) signals. Functional connectivity is separately measured at rest, during an attention task, and during a memory task. We assess these structural and functional measures within previously-identified resting-state functional networks, denoted task-positive and task-negative networks, that have been independently shown to be strongly anticorrelated at rest but also involve regions of the brain that routinely increase and decrease in activity during task-driven processes. We find that the density of anatomical connections within and between task-positive and task-negative networks is differentially related to strong, task-dependent correlations in neural activity. The space mapped out by the observed structure-function relationships is used to define a quantitative measure of separation between resting, attention, and memory states. We find that the degree of separation between states is related to both general measures of behavioral performance and relative differences in task-specific measures of attention versus memory performance. These findings suggest that the observed separation between cognitive states reflects underlying organizational principles of human brain structure and function.

## Author Summary

Human cognitive function is thought to be supported by patterns of correlated neural activity. While recent work has shown that such functional correlations are differentially supported by specific properties of anatomical brain connectivity, the extent to which brain anatomy shapes cognition is not understood. In this study, we develop new network-based approaches for relating anatomical connectivity, correlations in neural activity (functional connectivity), and behavioral task performance. We use noninvasive neuroimaging techniques to measure whole-brain connectivity in 71 subjects across three cognitive states: at rest, during an attention task, and during a memory task. By associating anatomical and functional connectivity with known functional brain networks, we show that the relative strength of inter- versus intra-network connectivity distinguishes between resting, attention, and memory states. When compared across subjects, we further show that the observed relationship between brain anatomy and function is predictive of individual differences in attention and memory task performance.

## Introduction

The brain is continually active, whether in a state of rest or during the performance of task-directed function. Despite predictions that resting-state neural activity would be noisy and unconstrained, the human brain has been shown to exhibit patterns of correlated neural activity even in the absence of any task-directed function [1], [2]. Such correlations in “default mode” activity, which have been consistently identified within a diffuse network of brain regions [3]–[6], are thought to support the functional organization of the brain [2].

Signatures of task-related function have similarly been identified in task-free states based on anticorrelations in spontaneous neural activity between default mode and task-related brain regions [7]–[10]. Together, these sets of brain regions have widely been associated with two functional networks, denoted task-positive and task-negative, composed of regions known to become more (task-positive) and less (task-negative) active during the task performance relative to their behavior at rest [7]. Correlations within these networks have been shown to support attention [11] and memory [12], [13] processes, and disruptions to these networks have been implicated in neurological disorders [14]–[17].

While such studies have characterized individual functional networks within single task domains, recent studies suggest that interactions between functional networks are important for shaping attention [18], memory [19], and motor learning [20], [21] performance. Anatomical studies have additionally shown that structural measures, such as the length and number of white matter tracts linking brain regions, play important roles in distinguishing global task-dependent changes in functional correlations [22]. Together, these findings suggest that anatomy may be important for shaping task-dependent interactions between functional networks, and these interactions may in turn be important for shaping behavior. Such relationships, however, are not understood. Does functional connectivity between networks vary systematically across across resting and task-driven cognitive states? To what extent is such variation differentially supported by underlying anatomical organization? How do such relationships between anatomy and function shape behavior?

To address these questions, we examine whether patterns of anatomical connectivity relate to the task-dependent strength of correlated neural activity within and between task-positive and task-negative networks, and we examine the extent to which such relationships are linked to behavioral differences in attention and memory performance. Structural and functional connectivity are estimated in 71 subjects using noninvasive neuroimaging techniques, where functional connectivity is separately estimated in three cognitive states: (*i*) at rest, (*ii*) during an attention task, and (*iii*) during a memory task. Behavioral performance is assessed in the same subjects during both the attention and memory tasks.

In what follows, we uncover task-dependent links between human brain anatomy, function, and behavior both within individual subjects and across groups of subjects. We group brain regions based on their involvement in the task-positive and task-negative networks defined in [7], and we show that the strength of anatomical connectivity within versus between these networks differentially supports strong, task-dependent functional correlations. The space mapped out by the observed structure-function relationships can be used to quantify a measure of separation between cognitive states, and we show that individual variability in this separation is linked to behavioral performance during both attention and memory tasks. Together, these results reveal that cognitive states are differentially supported by specific patterns of anatomical connectivity, and the observed association between anatomy and function is predictive of behavior.

## Methods

### Ethics Statement

Informed written consent was obtained from each subject prior to experimental sessions. All procedures were approved by the University of California, Santa Barbara Human Subjects Committee.

### Network Models of Brain Connectivity

We consider a complex network description of the human brain in which localized brain regions are represented as nodes, and the strengths of structural or functional connectivity between brain regions are represented as weighted, undirected connections between nodes [23], [24].

**Figure 1:**
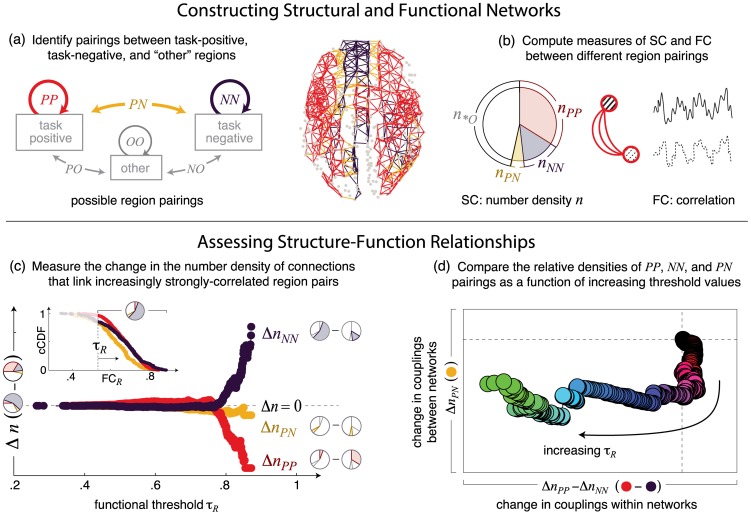
Identifying structure-function relationships in task-related networks. (a) In both representative and subject-specific brain networks, we identify brain regions that belong to the task-positive and task-negative network described in [7], and we label all remaining regions as “other” regions. There are six possible types of couplings between these three types of regions. We focus on three of these couplings: those between two task-negative regions (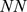), between two task-positive regions (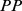), and between a task-positive and a task-negative region (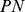). These couplings are highlighted in the axial view of the representative brain network. (b) We compute measures of structural (SC) and functional (FC) connectivity between each pair of regions by measuring the number of white matter streamlines linking two regions (SC) and the task-dependent strength of functional correlation between BOLD time series measured within regions (FC). The pie chart shows the decomposition of all structural connections into those that link two task-positive (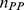), two task-negative (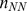), one task-positive and one task-negative (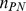), and all other regions (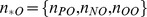). (c) We assess variations 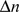 in these number densities as we bias toward increasingly strong functional correlations. This relationship is illustrated here for the representative brain network, where variations 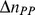, 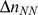, and 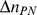 are shown as a function of the resting-state threshold 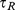. This can be understood as computing the change in composition of the pie chart shown in (b) while incrementally biasing toward strongly-correlated region pairs with functional correlations above the threshold value 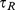. Inset: complementary cumulative distribution function (cCDF) of FC*_R_* computed for 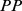, 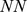, and 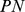 couplings, where the 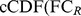) measures the probability of finding 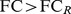 for every value of 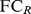. The variable threshold 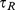 selects the subset of connections with 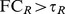. (d) The changes in 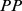, 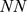, and 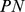 densities can be compactly represented by comparing the degree of within-network coupling, quantified by the relative change in 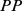 versus 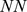 densities (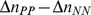), with the degree of between-network coupling, quantified by the change in 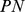 density (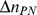). This representation reveals that strong resting-state FC is supported by strong local coupling within the task-negative network, represented by the increase in 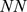 relative to 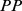 density, and weak coupling between task-positive and task-negative networks, represented by the decrease in 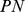 density.

A set of 600 cortical and subcortical regions roughly equal in size are chosen by upsampling anatomical regions within the Automated Anatomical Labeling (AAL) Atlas [25] (Text S1). We identify a total of 368 regions in our atlas that overlap wholly or partially with regions in task-positive and task-negative networks (Text S1), and we refer to the remaining regions as “other” regions. We focus on three of the six possible couplings between these three region types: couplings between two task-positive regions (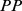), two task-negative regions (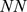), and one task-positive and one task-negative region (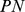). We then compare these couplings to the remaining set of couplings between task-positive and other regions (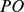), between task-negative and other regions (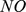), and between two other regions (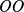). Figure 1a shows a schematic of possible couplings.

To construct brain networks from this set of regions, we weight connections between regions by measures of structural and functional connectivity. Structural connectivity (SC) is obtained from diffusion tensor imaging (DTI) measurements via a tractography algorithm used to identify the number of white matter streamlines linking two regions [22]. Functional connectivity (FC) is obtained from functional magnetic resonance imaging (fMRI) measurements by computing Pearson's correlations between regional mean blood oxygen level dependent (BOLD) time series. FC is separately estimated (*i*) at rest (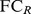), (*ii*) during the performance of an attention task (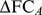), and (*iii*) during the performance of a memory task (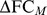) [26]. As task-based fluctuations in BOLD signals are small in comparison to resting-state values [27], task-based FC is computed in deviations 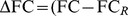) from rest [22]. See Text S1 and [22] for details regarding task design, connectivity estimates, and methodological considerations.

Each subject is described by a structural and functional brain network whose connections are weighted by subject-specific values of SC and FC. Group-level properties can similarly be described by a “representative” brain network that combines information across all subjects. We construct two representative networks, one structural and one functional, by averaging the corresponding sets of connection weights across subject-specific networks, such that the representative connection weights correspond to the group mean values of SC and FC, consistent with previous studies [22], [28]. Note that alternative techniques for constructing group-based connectivity networks may capture slightly different aspects of subject-specific network topology [29].

The assessment of group-level properties requires that we consider the degree to which SC is reliably present across subjects. While FC is typically non-sparse, SC can be both sparse and variable across subjects [22], [30]. We therefore restrict all subsequent analyses to the subset of region pairs that are consistently linked by one or more white matter streamlines in at least 80% of subjects. We confirm that the observed structural and functional properties of the set of thresholded connections are robust to our specific choice of thresholding values (Text S1).

## Results

### Couplings between Task-Positive and Task-Negative Networks Differentiate Cognitive States

Functional correlations within task-positive and task-negative networks have been separately linked to attention and memory processes, but the integrated function of these networks is, as of yet, unclear. Should we view these networks as distinct modules that compartmentalize function, or as integrated networks that couple together in a task-dependent manner to shape cognitive function?

To address this question, we examine the extent to which couplings within and between task-positive and task-negative networks differentially shape task-dependent distributions of functional connectivity. We first assess the distributions of resting-, attention-, and memory-state functional connectivity within the sets of 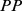, 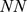, and 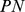 couplings. These distributions exhibit features that are consistent with known properties of task-positive and task-negative networks. In the resting state, both 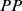 and 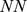 couplings exhibit stronger correlations than 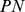 couplings (inset of Figure 1c), consistent with the definition of task-positive and task-negative networks based on strong correlations within each network and anticorrelations between networks [7] (note that anticorrelations are manifested here as weaker correlations between 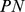 relative to 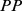 and 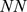 region pairs). Similarly, in the attention state, 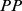 and 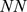 couplings respectively exhibit larger increases and decreases in FC relative to the distribution of 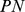 couplings (Text S1), consistent with known attention-driven increases and decreases in task-positive versus task-negative network activity.

Given that strong functional correlations are hypothesized to support state-dependent cognitive function, we focus our analysis on the strong values of FC within the leading edges of the resting-, attention-, and memory-state distributions. To isolate strong correlations in each cognitive state, we apply a variable threshold to each distribution of FC. The thresholding process, which selects connections above a specified threshold value of connectivity strength, is common to graph theoretic analyses [24] and can be used to assess network properties at a fixed threshold value (e.g. [28], [31]) or across variations in threshold values (e.g. [30], [32], [33]). Here, we apply a threshold to the distribution of FC, and we examine changes in network connectivity across variations in this threshold value. The use of a variable threshold enables us to isolate structural network properties that support increasingly strong functional correlations.

We characterize changes in network connectivity by examining the distribution of 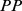, 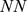, and 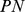 couplings that support functional correlations above a variable threshold 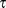. A given threshold value 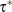 will select the set of region pairs with 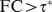. Within this set of region pairs, we measure the fractional number density 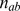 of all structural connections that couple a given pair of regions 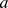 and 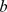, with region labels 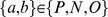. This process can be viewed as assessing the FC-dependent connectivity of a weighted structural network in which connections, defined by the reliable presence of SC in 80% of subjects, are weighted by the number of fiber tracts linking a given pair of regions. Qualitatively similar results are achieved by analyzing the connectivity of an unweighted structural network. However, we find that weighted networks better distinguish strong FC between different cognitive states than do unweighted networks, suggesting that both the presence and degree of structural connectivity play important roles in supporting strong state-dependent FC (see Text S1 for comparison of weighted and unweighted network analyses).

We vary the functional threshold 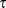 and compute the change 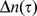 in number density relative to baseline (with baseline computed in the absence of any threshold). We find that the relative changes in 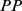, 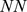, and 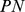 densities vary systematically across resting, attention, and memory states. In the resting state, for example, we find that strongly-correlated region pairs are supported by a high density of 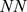 connections (positive 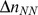) and a low density of 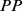 connections (negative 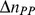) relative to their baseline values (Figure 1c).

To compare these relationships across cognitive states, we examine two quantities: the change in 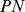 density (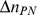) and the relative changes in 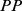 versus 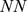 densities (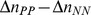). Both quantities are evaluated as a function of the task-dependent thresholds 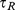, 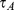, and 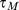. The quantity 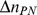 measures the degree of coupling between task-positive and task-negative networks, with positive (negative) values of 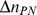 indicating increased (decreased) coupling relative to baseline. In comparison, the quantity 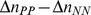 measures the degree of localized coupling within either the task-positive or task-negative network, with positive (negative) values indicating localized coupling within the task-positive (task-negative) network.

Figure 1d illustrates the relationship between these two quantities in the resting state (note that this is a condensed representation of the information shown in Figure 1c). We refer to this representation as a “state-space” mapping, as it enables us to isolate the structure-function relationships that characterize each cognitive state.

**Figure 2:**
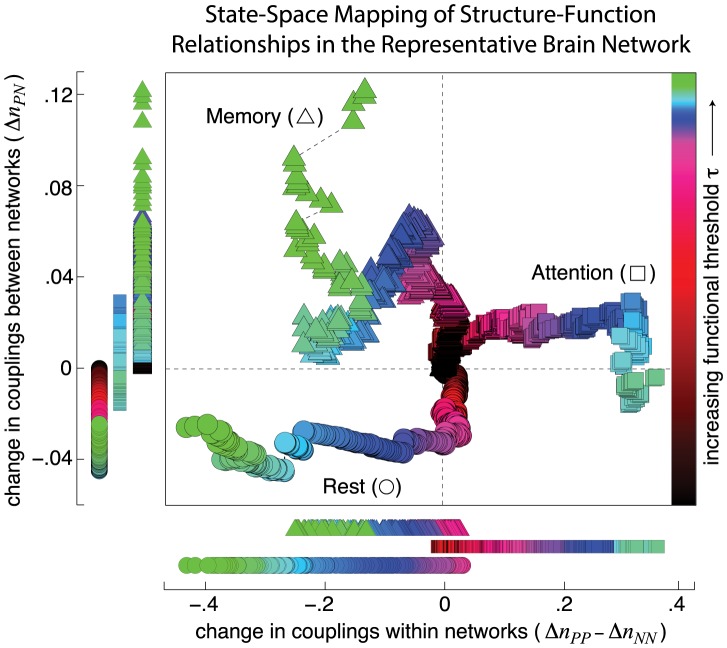
State-space mapping of structure-function relationships. Density of between-network couplings (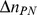) versus within-network couplings (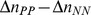) are shown as a function of the increasing functional threshold 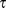 in the representative brain network for the resting (circular markers), attention (square markers), and memory (triangular markers) states. Comparison of these network couplings reveals a large degree of separation between rest, attention, and memory states, with the degree of separation increasing as a function of 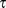. The resting state is characterized by an increased density of 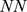 relative to 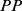 connections and a decreased density of 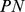 contributions. In comparison, the attention state is characterized by an increased density of 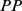 relative to 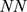 connections. The memory state shares features of both the resting and attention states, showing an increased density of 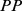 relative to 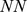 connections and an increased density of 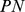 connections.

Comparison across cognitive states reveals that resting, attention, and memory states occupy distinct regions of this state space, differing from one another in the types of connection densities that support strong functional correlations (Figure 2). Strong resting-state correlations are supported by a decreased density of 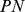 connections and an increased density of 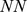 relative to 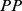 connections, reflecting strong localization within the task-negative network and weak coupling between the task-positive and task-negative networks. Strong attention-state correlations, in comparison, are supported by an increased density of 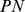 connections and an increased density of 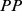 relative to 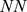 connections, reflecting strong localization with the task-positive network and strong coupling between the task-positive and task-negative networks. Finally, strong memory-state correlations share features of both attention and rest, as they are supported by an increased density of 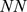 relative to 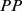 connections and an increased density of 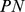 connections.

These findings are consistent with known properties of task-positive and task-negative networks. The anticorrelation between the task-positive and task-negative networks manifests here as a decreased density of 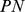 connections supporting strong resting-state correlations. Similarly, the known importance of task-positive brain regions in attention tasks manifests here as an increased density of 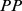 relative to 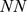 connections supporting strong attention-state correlations. Lastly, memory-state functional networks are known to overlap with both resting- and attention-state functional networks [13]. We similarly find that the types of connection densities that support strong memory-state FC are similar to those connection densities that support strong resting- and attention-state FC.

Together, these results show that structural connections between task-related functional networks distinguish strong functional correlations measured in different cognitive states.

### Individuals Can Be Grouped Based on Similarities in State-Space Relationships

The state-space description shown in Figure 2 reveals that cognitive states differ from one another in the structural features that support strong functional correlations. We investigate whether the observed separation between cognitive states, as quantified by differences in such structure-function relationships, is a general feature of subject-specific networks.

**Figure 3:**
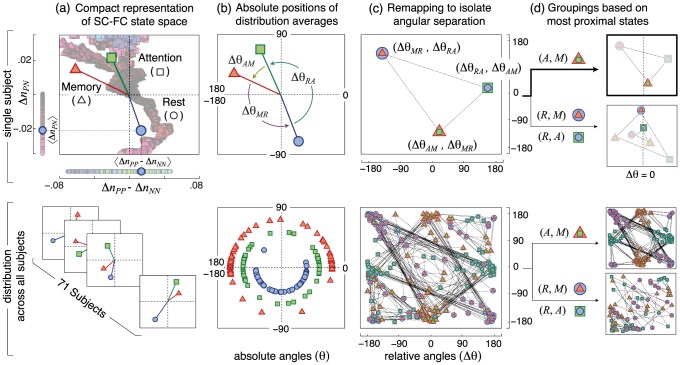
Individual variability in state-space relationships. Subject-specific relationships between resting (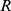), attention (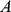), and memory (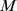) states shown for a single subject (upper row) and for the entire set of subjects (lower row). (a) Subject-specific brain networks can each be described by a state-space of network couplings, quantified by 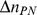 versus 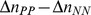, that is analogous to the state space shown in Figure 2 for the representative brain network. Each subject can then be compactly described by a triad of points, one each for resting (circular marker), attention (square marker), and memory (triangular marker) states, that marks the distribution averages 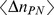 and 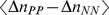 for each cognitive state. (b) The separation between two states can be quantified by the angular separation 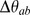 between distribution averages, with 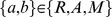. (c) To isolate the angular separation between states, we perform a remapping of the state space in which we represent each individual by a triangle whose vertices are defined by cyclical permutations of 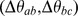. Each vertex is visually indicated by the superposition of markers that denote the two cognitive states related by 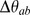 (e.g. 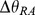 is denoted by the superposition of a circular (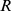) and square (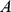) marker). This remapping reveals a high degree of inter-subject consistency in the relative separation between states, as noted by the clusters of markers of a given type and the highly overlapping triangles linking these clusters. (d) Subjects can be grouped according to the rank order of angular separations. This method naturally isolates one primary group of subjects who show the smallest separation between attention and memory states. The subject shown in the upper row falls into this primary group, as indicated by the proximity of 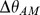 (superposition of triangular and square markers) to the vertical dotted line marking 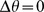. The remaining two secondary groups show the smallest separation between rest and memory (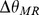 closest to 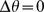) and between attention and memory (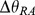 closest to 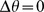).

Each subject-specific brain network can be remapped onto a state-space, analogous to that shown in Figure 2, that compares subject-specific values 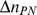 and 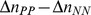 across resting, attention, and memory states. To compare across subjects, we compactly represent each subject by a triad of resting- (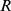), attention- (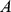), and memory-state (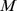) distribution averages of 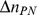 and 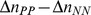 (Figure 3a). The degree of separation between cognitive states can then be quantified by the angular separation 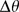 between distribution averages.

To isolate inter-subject variations in angular separation, we perform a remapping of the state space in which we represent each individual by a triangle whose vertices are defined by cyclical permutations of 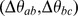, where 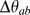 quantifies the angular separation between states 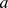 and 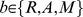 (Figure 3b). In this representation, the size of a given triangle captures the degree of symmetric separation between cognitive states, with smaller triangles indicating that states are separated from one another by nearly equal angular distances. Similarly, the rotation of a given triangle captures the rank-ordering of angular separations.

This remapping, shown in Figure 3b–c, reveals that the degree of separation between cognitive states is highly consistent across subjects. This consistency is illustrated by the clustering of points of the same color, and similarly by the largely overlapping sets of triangles that link these clusters. In a majority of subjects, attention and memory occupy similar regions of state space, as quantified by small values of 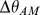 and as illustrated by the clustering of green points near the center vertical axis. In these same subjects, rest occupies a distinct region of state space far from both attention and memory, as quantified by large values of 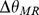 and 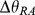 and as illustrated by the clustering of blue and red points near the left and right vertical axes. The observed organization is not an artifact of our analysis techniques, as confirmed via comparison with a null model in which “task-positive,” “task-negative,” and “other” region labels are randomly reassigned (Text S1).

This representation naturally organizes subjects into three distinct groups based on the relative degree of separation between cognitive states (Figure 3d). The primary group (66% of subjects) exhibits less separation between the two task states than between task and resting states. These separations indicate that similar structural connections support large changes in both attention and memory FC, and these structural connections differ from those that support strong resting-state FC. The remaining subjects comprise two secondary groups, the first exhibiting the least separation between resting and memory states (23% of subjects), and the second exhibiting the least separation between resting and attention states (11% of subjects). Small separations between resting and task states indicate that task-dependent changes in FC, measured either during attention or memory tasks, are supported by similar structural connections as those that support strong resting-state FC.

Importantly, the primary and secondary groups identified here are statistically similar to those groups identified from clustering algorithms (Text S1), confirming that the separation between cognitive states captures communities of subjects with similar structure-function relationships. However, the methodology developed here differs from such data-driven clustering algorithms in that it provides an intuitive framework for understanding relationships between cognitive states based on similarities in the underlying structural features that support these states.

### State-Space Relationships Predict Deviations in Behavioral Performance

The organization of subjects based on the separation between cognitive states raises two important questions about the potential relationships between structure, function, and behavior. First, do primary and secondary groups, as identified by the structure-function relationships that distinguish between cognitive states, show absolute differences in attention and memory performance? Second, is the degree of separation between attention and memory states, being the property that distinguishes between primary and secondary groups, indicative of relative differences between attention and memory performance?

We address both questions by comparing the behavioral performance of subjects within the primary versus secondary groups. We first assess whether the secondary groups, being outliers in the state-space mapping of structure-function relationships, are also outliers in absolute measures of attention and memory performance. We then assess whether the secondary groups, showing larger separations between attention and memory states in the state-space mapping, also show larger differences in attention versus memory performance.

Both the attention and memory tasks were designed to measure subjects' ability to flexibly switch between decision strategies based on probabilistic information about the (*i*) likely position of a visual cue to be identified during the attention task, or (*ii*) the likelihood that a visual cue had been previously presented during a memory task [22], [26].

Based on the design of these tasks, we focus on three relevant measures of task performance: the criterion switch score (CS), the d-prime (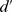) score, and the average reaction time (RT). CS measures strategic flexibility in switching between decision making strategies, with higher values indicating the ability to more readily switch strategies. The measure 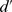 assesses perceptual (attention task) or mnemonic (memory task) sensitivity as related to accuracy, with higher values indicating higher sensitivity and therefore higher accuracy. Lastly, average RT measures the average time between the appearance of a stimulus and a subject's response (via a button press) to that stimulus. A more detailed discussion of these performance measures can be found in [26].

**Figure 4:**
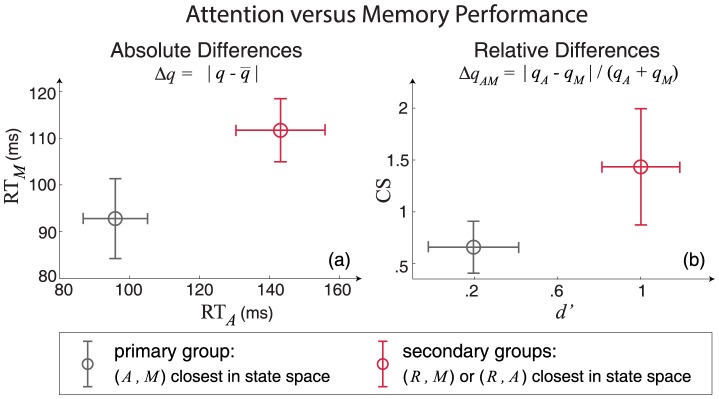
Behavioral performance of primary versus secondary groups. Absolute (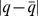) and relative (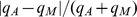) differences in performance measures 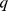 for attention (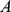) and memory (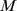) tasks, where 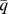 denotes the group average value of 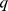. Performance differences are reported as means and standard errors for subjects within the primary (gray) and secondary (red) groups. (a) The secondary groups show larger absolute differences in reaction time (RT) for both attention and memory tasks. (b) The secondary groups show larger relative differences between attention and memory tasks for both the criterion switch score (CS) and the d-prime (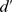) score.

We assess absolute performance by computing the deviation 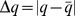 of a given measure 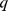 from the group average value 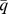. We find that the secondary groups, being outliers by the measure of relative separation between cognitive states, are also outliers in attention and memory RT (Figure 4a). The observed difference between primary and secondary groups is statistically significant (a one-tailed 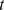-test of 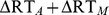 gives 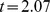, 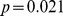). We similarly assess relative performance by computing the relative difference 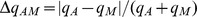 between a given measure assessed during attention (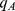) versus memory (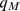) tasks. We find that the secondary groups, exhibiting larger separations between attention and memory states, also show larger differences between the values of 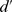 and CS measured during attention versus memory tasks (Figure 4b). The observed difference between primary and secondary groups is again statistically significant (a one-tailed 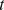-test of 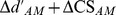 gives 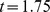, 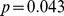). Note that 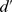 and CS have previously been shown to be strongly correlated with one another [26], such that relative differences in one measure may drive relative differences in the other. A repeated measures analysis of variance (ANOVA; with two state-space groupings, primary and secondary, as categorical measures and with nine behavioral variables [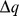 and 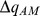 for 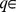 {RT, CS, 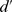}] as repeated measures) further confirms that the observed differences between primary and secondary groups are statistically significant, with a main effect of grouping of 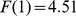 and 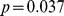 (see Text S1 for full table of ANOVA results).

These results show that the observed separations between cognitive states, which arise from differences in the patterns of structural connectivity that support strong functional correlations, are linked to both absolute differences in overall performance and relative differences between attention and memory performance. Interestingly, the primary and secondary groups differ from one another in absolute measures of RT but relative measures of 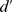 and CS. Given that RT is a more general performance measure, while 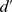 and CS are targeted by the attention and memory tasks under consideration, this suggests that individual differences in the rank-ordering of separations between cognitive states are manifested in general measures of performance, while individual differences in the degree of separation between two states are manifested in task-specific measures pertaining to those states.

## Discussion

Anatomical connectivity plays an important role in shaping human cognitive function. To systematically probe relationships between neural anatomy, function, and behavior, we examine structural and functional connectivity within and between two functional networks, task-positive and task-negative, that have been implicated in a wide range of task-free and task-driven studies of brain function [7], [9], [11]–[13]. We develop an intuitive framework for understanding how structural connectivity within and between these networks shapes task-dependent correlations in neural activity. Within this framework, cognitive states can be characterized by patterns of structural connectivity that support strong functional correlations. When examined across many subjects, differences between cognitive states are linked to differences in behavioral task performance.

We find that the relative number of structural connections between task-positive and task-negative networks differentially supports strong, task-dependent functional connectivity, suggesting that task performance modulates interactions within task-based networks by altering the strength of the strongest interactions. The finding that strong resting-state FC is supported by increased coupling within the task-negative network but decreased coupling between the task-positive and task-negative networks supports the view of default mode function described in a wide range of studies [2], [5]–[7]. Similarly, the increased coupling within the task-positive network during attention and within the task-negative network during memory supports previously identified task-positive regions important for attention [7] and task-negative regions important for memory [12], [13]. It has additionally been suggested that the task-positive network can be decomposed into three subnetworks [10], and separate examination of structural and functional features within these subnetworks may further delineate the structure-function relationships that distinguish between different task states.

The space mapped out by couplings within and between the task-positive and task-negative networks can be used to assign a quantitative measure to the separation between resting, attention, and memory states. When compared across subjects, we find a high degree of consistency in the relative separation between states, with a majority of subjects showing small separations between attention and memory states but large separations in these two states from rest. This suggests that, within individual subjects, strong task-dependent changes in FC are supported by similar patterns of SC.

We do find subjects who deviate from this pattern of organization, exhibiting large separations between attention and memory states. Such large separations indicate that changes in the strength of functional correlations associated with attention versus memory tasks are supported by different patterns of structural connectivity. The deviation of these subjects from the majority, as measured by structural and functional connectivity, has two important consequences for behavioral performance. First, the subjects who differ from the majority in relative separation between cognitive states also differ from the majority in general measures of behavioral performance. Second, the way in which these subjects differ from the majority, namely by exhibiting larger separations between attention and memory states, matches the way in which they differ from the majority in behavioral performance, in that they exhibit larger relative differences in task-specific measures of attention and memory performance. These findings suggest that the overall organization of rest and task states is predictive of general performance, while the degree of separation between two task states is predictive of relative differences in performance between the two tasks.

These findings build upon known structure-function relationships in the human brain. Previous work has shown that structural properties shape global patterns of functional connectivity in a task-dependent manner [22]. Our results show that these global changes are modulated by regional changes in connectivity patterns within and between functional networks. These findings are compatible with previous studies of default-mode (task-negative) and fronto-parietal (task-positive) networks, which found increases in intra-network connectivity during memory tasks relative to rest, and increases in inter-network connectivity with memory load [34]. However, our results provide a modified view of the role of network connectivity: within the set of strongest connections, relative differences between inter- versus intra-network connectivity not only distinguish between cognitive states, but they also predict individual performance differences within and across tasks. A growing number of recent studies have found similar links between dynamic changes in functional connectivity and behavioral performance within single task domains [18]–[21]. This study builds on these ideas by examining the degree to which state-dependent changes in connectivity, characterized by a measure of separation between cognitive states, is predictive of state-dependent behavior. Together, these findings suggest an important role for dynamic reconfiguration of brain networks not only within individual cognitive states, as has been observed previously, but also across different states.

### Methodological Considerations

This work examined couplings within and between the task-positive and task-negative networks identified in [7]. However, a wide range of different functional networks have been identified in the human brain and have been linked to both resting-state and task-driven neural activity (e.g. [10], [35], [36]). A more detailed comparison of such functional networks could provide further insight into the structure-function relationships that distinguish between different cognitive states. Furthermore, interactions within and between these networks have been shown to change throughout development [37], [38] and aging [39], suggesting that the state-space of relationships found here could exhibit different characteristics across subjects of different ages.

We demonstrated that patterns of structural connectivity within and between task-positive and task-negative networks differentially support task-dependent FC. The observed separation between cognitive states was measured as a function of the relative number of structural connections linking a given region pair, but qualitatively consistent results were observed when structural connectivity was defined in a binary manner based on the reliable presence of structural connections across a majority of subjects (Text S1). Probabilistic tractography algorithms (e.g. [40]), which can identify crossing or branching fibers that would not be identified by the deterministic tractography algorithms used here, could improve estimates of structural connectivity and are therefore expected to further strengthen these results. Recent advances in computational platforms (e.g. [41]–[43]) provide additional model-based approaches for simulating brain dynamics using subject-specific patterns of anatomical connectivity. These platforms enable the identification of spatiotemporal motifs that support cognitive activity, as well as the biophysical parameters that constrain these motifs. Such anatomically-informed modeling approaches might help isolate features of structural brain architecture that shape the state-space mapping described here, such as transmission delays induced by long fiber tracts, or signal amplification due to large fiber bundles. Furthermore, such approaches might identify additional network motifs that distinguish state-dependent cognitive function. In combination with subject-specific anatomical constraints, these methods could help elucidate how individual variability in anatomical connectivity constrains functional interactions to ultimately shape behavioral performance.

In assessing state-space relationships, resting, attention, and memory functional scans were taken to represent individual cognitive states. However, there is evidence of spatial and temporal variability within single functional domains [44]–[47], suggesting an interplay between multiple cognitive states that each become more or less active throughout the duration of a given scan. The framework presented here, when combined with approaches for assessing nonstationary correlational structure [48], could help uncover structural features that distinguish different dynamical patterns of activity observed within a given functional domain.

### Final Remarks

The observation that structure-function relationships between cognitive states exhibit common state-space features suggests that these features may reflect general organizational principles of the brain. The state-space representation may therefore be useful for defining normative bounds on large-scale patterns of brain organization. When the features of this space are probed using suitably large numbers of subjects, regions of this space not occupied by healthy individuals could be predictive of disrupted structural or functional connectivity. A further characterization of the observed structure-function relationships across different behavioral and genetic measures could potentially be used to develop objective diagnostic measures of disrupted functionality.

## Supporting Information

Text S1We first describe the experimental methods and participants of this study. We then describe the construction of task-positive and task-negative networks, and we highlight the specific anatomical regions of the brain involved in the task-related network couplings 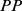, 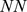, 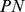, and 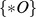. We then show the distributions of resting- (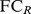), attention- (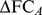), and memory-state (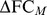) functional correlations between 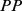, 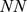, and 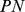 region pairs. By thresholding these distributions of FC, we show the resulting variations 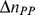, 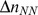, and 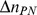 in the number density of structural connections linking strongly-correlated region pairs. Together, these distributions of 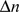 were used to define the state-space mapping of resting, attention, and memory states shown in Figure 2 of the main text. We show the observed separation between cognitive states is robust to the specific choices made in constructing the representative brain network and in thresholding the resulting distributions of FC. When compared across subjects, this state-space mapping revealed significant inter-subject organization in the relative separations between cognitive states, with subjects naturally organizing into primary and secondary groups (Figure 3 of the main text). We show that the observed organization into such groups is not an artifact of our specific analysis techniques, and we confirm that the primary and secondary groups are statistically similar to the groups identified by a clustering algorithm. Lastly, these groups were shown to exhibit significant differences in behavioral task performance (Figure 4 of the main text). We describe the full set of ANOVA results that were used to confirm the statistical significance of these performance differences.(PDF)

## References

[1] Raichle ME, MacLeod AM, Snyder AZ, Powers WJ, Gusnard DA, et al (2001) A default mode of brain function. Proc Natl Acad Sci USA 98: 676–682. 11209064 10.1073/pnas.98.2.676 PMC14647

[2] Fox MD, Raichle ME (2007) Spontaneous fluctuations in brain activity observed with functional magnetic resonance imaging. Nat Rev Neurosci 8: 700–711. 17704812 10.1038/nrn2201

[3] Greicius MD, Krasnow B, Reiss AL, Menon V (2003) Functional connectivity in the resting brain: a network analysis of the default mode hypothesis. Proc Natl Acad Sci U S A 100: 253–258. 12506194 10.1073/pnas.0135058100 PMC140943

[4] Damoiseaux JS, Rombouts SARB, Barkhof F, Scheltens P, Stam CJ, et al (2006) Consistent resting-state networks across healthy subjects. Proc Natl Acad Sci USA 103: 13848–13853. 16945915 10.1073/pnas.0601417103 PMC1564249

[5] Long XY, Zuo XN, Kiviniemi V, Yang Y, Zou QH, et al (2009) Default mode network as revealed with multiple methods for resting-state functional mri analysis. J Neurosci Methods 171: 349–355. 18486233 10.1016/j.jneumeth.2008.03.021

[6] Shehzad Z, Kelly AMC, Reiss PT, Gee DG, Gotimer K, et al (2009) The resting brain: Uncon-strained yet reliable. Cereb Cortex 19: 349–355. 19221144 10.1093/cercor/bhn256 PMC3896030

[7] Fox MD, Snyder AZ, Vincent JL, Corbetta M, Essen DCV, et al (2005) The human brain is intrinsically organized into dynamic, anticorrelated functional networks. Proc Natl Acad Sci USA 102: 9673–9678. 15976020 10.1073/pnas.0504136102 PMC1157105

[8] Fox MD, Corbetta M, Snyder AZ, Vincent JL, Raichle ME (2006) Spontaneous neuronal activity distinguishes human dorsal and ventral attention systems. Proc Natl Acad Sci USA 103: 10046–10051. 16788060 10.1073/pnas.0604187103 PMC1480402

[9] Seeley WW, Menon V, Schatzberg AF, Keller J, Glover GH, et al (2007) Dissociable intrinsic connectivity networks for salience processing and executive control. J Neurosci 27: 2349–2356. 17329432 10.1523/JNEUROSCI.5587-06.2007 PMC2680293

[10] Power JD, Cohen AL, Nelson SM, Wig GS, Barnes KA, et al (2011) Functional network organization of the human brain. Neuron 72: 665–678. 22099467 10.1016/j.neuron.2011.09.006 PMC3222858

[11] Mennes M, Kelly C, Zuo XN, Martino AD, Biswal BB, et al (2011) Inter-individual differences in resting-state functional connectivity predict task-induced bold activity. NeuroImage 50: 1690–1701. 20079856 10.1016/j.neuroimage.2010.01.002 PMC2839004

[12] Hampson M, Driesen NR, Roth JK, Gore JC, Constable RT (2010) Functional connectivity between task-positive and task-negative areas and its relation to working memory performance. Magnetic Resonance Imaging 28: 1051–1057. 20409665 10.1016/j.mri.2010.03.021 PMC2936669

[13] Kim H, Daselaar SM, Cabeza R (2010) Overlapping brain activity between episodic memory encoding and retrieval: Roles of the task-positive and task-negative networks. NeuroImage 49: 1045–1054. 19647800 10.1016/j.neuroimage.2009.07.058 PMC2764805

[14] Greicius MD, Supekar K, Menon V, Dougherty RF (2009) Resting-state functional connectivity reects structural connectivity in the default mode network. Cereb Cortex 19: 72–78. 18403396 10.1093/cercor/bhn059 PMC2605172

[15] Broyd SJ, Demanuele C, Debener S, Helps SK, James CJ, et al (2009) Default-mode brain dysfunction in mental disorders: A systematic review. Neurosci, Biobehav Rev 33: 279–296. 18824195 10.1016/j.neubiorev.2008.09.002

[16] Kennedy DP, Courchesne E (2008) The intrinsic functional organization of the brain is altered in autism. NeuroImage 39: 1877–1885. 18083565 10.1016/j.neuroimage.2007.10.052

[17] Liu H, Kaneko Y, Ouyang X, Li L, Hao Y, et al (2012) Schizophrenic patients and their unaffected siblings share increased resting-state connectivity in the task-negative network but not its anticorrelated task-positive network. Schizoph bull 38: 285–294. 20595202 10.1093/schbul/sbq074 PMC3283150

[18] Daitch AL, Sharma M, Roland JL, Astafiev SV, Bundy DT, et al (2013) Frequency-specific mechanism links human brain networks for spatial attention. Proc Natl Acad Sci USA 110: 19585–19590. 24218604 10.1073/pnas.1307947110 PMC3845177

[19] Fornito A, Harrison BJ, Zalesky A, Simons JS (2012) Competitive and cooperative dynamics of large-scale brain functional networks supporting recollection. Proc Natl Acad Sci USA 109: 12788–12793. 22807481 10.1073/pnas.1204185109 PMC3412011

[20] Bassett DS, Wymbs NF, Porter MA, Mucha PJ, Carlson JM, et al (2011) Dynamic reconfiguration of human brain networks during learning. Proc Natl Acad Sci USA 108: 7641–7646. 21502525 10.1073/pnas.1018985108 PMC3088578

[21] Bassett DS, Wymbs NF, Rombach MP, Porter MA, Mucha PJ, et al (2013) Task-based coreperiphery organization of human brain dynamics. PLoS Comput Biol 9: e1003171. 24086116 10.1371/journal.pcbi.1003171 PMC3784512

[22] Hermundstad AM, Bassett DS, Brown KS, Aminoff EM, Freeman S, et al (2013) Structural foundations of resting-state and task-based neural activity in the human brain. Proc Natl Acad Sci USA 10.1073/pnas.1219562110 PMC3625268 23530246

[23] Bullmore E, Sporns O (2009) Complex brain networks: graph theoretical analysis of structural and functional systems. Nat Rev Neurosci 10: 186–198. 19190637 10.1038/nrn2575

[24] Bullmore E, Bassett DS (2011) Brain graphs: graphical models of the human brain connectome. AR Clinical Psychol 7: 113–140. 10.1146/annurev-clinpsy-040510-143934 21128784

[25] Tzourio-Mazoyer, Landeau B, Papathanassiou D, Crivello F, Etard O, et al (2002) Automated anatomical labeling of activations in spm using a macroscopic anatomical parcellation of the mni mri single-subject brain. NeuroImage 15: 273–289. 11771995 10.1006/nimg.2001.0978

[26] Aminoff EM, Clewett D, Freeman S, Frithsen A, Tipper C, et al (2012) Individual differences in shifting decision criterion: A recognition memory study. Mem Cogn 40: 127–134. 10.3758/s13421-012-0204-6 22555888

[27] Raichle ME, Minturn MA (2006) Brain work and brain imaging. Annu Rev Neurosci 29: 449–476. 16776593 10.1146/annurev.neuro.29.051605.112819

[28] Achard S, Salvador R, Whitcher B, Suckling J, Bullmore E (2006) A resilient, low-frequency, small-world human brain functional network with highly connected association cortical hubs. J Neurosci 26: 63–72. 16399673 10.1523/JNEUROSCI.3874-05.2006 PMC6674299

[29] Simpson S, Moussa M, Laurienti P (2012) An exponential random graph modeling approach to creating group-based representative connectivity networks. NeuroImage 60: 1117–1126. 22281670 10.1016/j.neuroimage.2012.01.071 PMC3303958

[30] Bassett DS, Brown JA, Deshpande V, Carlson JM, Grafton ST (2011) Conserved and variable architecture of human white matter connectivity. NeuroImage 54: 1262–1279. 20850551 10.1016/j.neuroimage.2010.09.006

[31] He Y, Chen Z, Evans A (2007) Small-world anatomical networks in the human brain revealed by cortical thickness from mri. Cereb Cortex 17: 2407–2419. 17204824 10.1093/cercor/bhl149

[32] Archard S, Bullmore E (2007) Efficiency and cost of economical brain functional networks. PLoS Comput Biol 3: e17. 17274684 10.1371/journal.pcbi.0030017 PMC1794324

[33] Bassett D, Bullmore E, Verchinski B, Mattay V, Weinberger D, et al (2008) Hierarchical organization of human cortical networks in health and schizophrenia. J Neurosci 28: 9239–9248. 18784304 10.1523/JNEUROSCI.1929-08.2008 PMC2878961

[34] Repovs G, Barch DM (2009) Working memory related brain network connectivity in individuals with schizophrenia and their siblings. Front Hum Neurosci 6: 137. 22654746 10.3389/fnhum.2012.00137 PMC3358772

[35] Dosenbach NU, Visscher KM, Palmer ED, Miezin FM, Wenger KK, et al (2006) A core system for the implementation of task sets. Neuron 50: 799–812. 16731517 10.1016/j.neuron.2006.04.031 PMC3621133

[36] Dosenbach NUF, Fair DA, Miezin FM, Cohen AL, Wenger KK, et al (2007) Distinct brain networks for adaptive and stable task control in humans. Proc Natl Acad Sci USA 104: 11073–11078. 17576922 10.1073/pnas.0704320104 PMC1904171

[37] Fair DA, Cohen AL, Power JD, Dosenbach NUF, Church JA, et al (2009) Functional brain networks develop from a “local to distributed” organization. PloS Comput Biol 5: e1000381. 19412534 10.1371/journal.pcbi.1000381 PMC2671306

[38] Supekar K, Musen M, Menon V (2009) Development of large-scale functional brain networks in children. PloS Biol 7: e1000157. 19621066 10.1371/journal.pbio.1000157 PMC2705656

[39] Beason-Held LL RS Kraut MA (2009) Stability of default-mode network activity in the aging brain. Brain Imaging Behav 3: 123–131. 19568331 10.1007/s11682-008-9054-z PMC2703608

[40] Parker GJ, Alexander DC (2003) Probabilistic Monte Carlo based mapping of cerebral connections utilising whole-brain crossing fibre information. Information Processing in Medical Imaging 18: 684–695. 15344498 10.1007/978-3-540-45087-0_57

[41] Knock S, McIntosh A, Kotter OSR, Hagmann P, Jirsa V (2009) The effects of physiologically plausible connectivity structure on local and global dynamics in large scale brain models. J Neurosci Methods 183: 86–94. 19607860 10.1016/j.jneumeth.2009.07.007

[42] Leon PS, Knock SA, Woodman MM, Domide L, Mersmann J, et al (2013) The virtual brain: a simulator of primate brain network dynamics. Front Neuroinform 7: 1–23. 23781198 10.3389/fninf.2013.00010 PMC3678125

[43] Ritter P, Schirner M, McIntosh A, Jirsa V (2013) The virtual brain integrates computational modeling and multimodal neuroimaging. Brain Conn 3: 121–145. 10.1089/brain.2012.0120 PMC3696923 23442172

[44] Chang C, Glover GH (2010) Time-frequency dynamics of resting-state brain connectivity measured with fmri. NeuroImage 50: 81–98. 20006716 10.1016/j.neuroimage.2009.12.011 PMC2827259

[45] Kang J, Wang L, Yan C, Wang J, Liang X, et al (2011) Characterizing dynamic functional con-nectivity in the resting brain using variable parameter regression and kalman filtering approaches. NeuroImage 56: 1222–1234. 21420500 10.1016/j.neuroimage.2011.03.033

[46] Kiviniemi V, Vire T, Remes J, Elseoud A, Starck T, et al (2011) A sliding time-window ica reveals spatial variability of the default mode network in time. Brain Connect 1: 339–347. 22432423 10.1089/brain.2011.0036

[47] Allen EA, Damaraju E, Plis SM, Erhardt EB, Eichele T, et al (2012) Tracking whole-brain connectivity dynamics in the resting state. Cereb Cortex 24 (3) 663–76. 23146964 10.1093/cercor/bhs352 PMC3920766

[48] Liu X, Duyn JH (2013) Time-varying functional network information extracted from brief instances of spontaneous brain activity. Proc Natl Acad Sci USA 110: 4392–4397. 23440216 10.1073/pnas.1216856110 PMC3600481

