# Supplementary material for: Structurally-Constrained Relationships between Cognitive States in the Human Brain
Source: PLoS Comput Biol. 2014 May 15;10(5):e1003591. doi: 10.1371/journal.pcbi.1003591 (PMC4022461; doi:10.1371/journal.pcbi.1003591)
Supplement: Text S1 — We first describe the experimental methods and participants of this study. We then describe the construction of task-positive and task-negative networks, and we highlight the specific anatomical regions of the brain involved in the task-related network couplings , , , and . We then show the distributions of resting- (), attention- (), and memory-state () functional correlations between , , and region pairs. By thresholding these distributions of FC, we show the resulting variations , , and in the number density of structural connections linking strongly-correlated region pairs. Together, these distributions of were used to define the state-space mapping of resting, attention, and memory states shown in Figure 2 of the main text. We show the observed separation between cognitive states is robust to the specific choices made in constructing the representative brain network and in thresholding the resulting distributions of FC. When compared across subjects, this state-space mapping revealed significant inter-subject organization in the relative separations between cognitive states, with subjects naturally organizing into primary and secondary groups (Figure 3 of the main text). We show that the observed organization into such groups is not an artifact of our specific analysis techniques, and we confirm that the primary and secondary groups are statistically similar to the groups identified by a clustering algorithm. Lastly, these groups were shown to exhibit significant differences in behavioral task performance (Figure 4 of the main text). We describe the full set of ANOVA results that were used to confirm the statistical significance of these performance differences. (PDF) [file pcbi.1003591.s001.pdf]

Structurally-constrained relationships between cognitive states in the human brain

A. M. Hermundstad[1,2], K. S. Brown[3,4,5,6], D. S. Bassett[1,7,8,9], E. M. Aminoff[10], A. Frithsen[11], A. Johnson[11], C. M. Tipper[12], M. B. Miller[11], S. T. Grafton[11] and J. M. Carlson[1]

[1]Department of Physics, University of California, Santa Barbara, CA USA
[2]Department of Physics and Astronomy, University of Pennsylvania, Philadelphia, PA USA
[3]Department of Biomedical Engineering, University of Connecticut, Storrs, CT USA
[4]Department of Physics, University of Connecticut, Storrs, CT USA
[5]Department of Chemical and Biomolecular Engineering, University of Connecticut, Storrs, CT USA
[6]Department of Marine Sciences, University of Connecticut, Groton, CT USA
[7]Sage Center for the Study of the Mind, University of California, Santa Barbara, CA USA
[8]Department of Bioengineering, University of Pennsylvania, Philadelphia, PA USA
[9]Department of Electrical and Systems Engineering, University of Pennsylvania, Philadelphia, PA USA
[10]Center for the Neural Basis of Cognition, Carnegie Mellon University, Pittsburgh, PA USA
[11]Department of Psychological and Brain Sciences, University of California, Santa Barbara, CA USA
[12]Department of Psychiatry, Faculty of Medicine, University of British Columbia, Vancouver, BC Canada

March 26, 2014

Text S1

CONTENTS

Summary **3**

Materials and Methods **4**
- Attention and Memory Task Design . . . 4
  - Attention Task . . . 4
  - Memory Tasks . . . 4
- Connectivity Estimates . . . 5
  - Structural Connectivity . . . 5
  - Functional Connectivity . . . 5

Results **5**
- Brain Regions Involved in Network Couplings . . . 5
  - Labeling of the Anatomical AAL Atlas . . . 6
  - Upsampling to the Uniform-600 Atlas . . . 6
- Contributions of Network Couplings to Strong FC . . . 6
- Robustness to Analysis Variations . . . 7
  - Thresholding . . . 7
  - Weighted versus Unweighted Network Representations . . . 8
- Comparisons of State-Space Mappings with Null Model . . . 8
- Algorithmic Detection of Community Structure in State-Space Mappings . . . 9
- ANOVA Results . . . 10

LIST OF TABLES

S1 Region Label Assignment . . . 7
S2 Repeated Measures ANOVA Results: Original Data . . . 11
S3 Repeated Measures ANOVA Results: Null Model . . . 11

LIST OF FIGURES

S1 Brain Regions Involved in Network Couplings . . . 12
S2 Contributions of Network Couplings to Strong Functional Connectivity . . . 13
S3 Robustness of State-Space Mapping to Thresholding . . . 14
S4 Robustness of State-Space Mapping to Weighted versus Unweighted Network Representations . . . 15
S5 Comparison of Individual State-Space Relationships to Null Model . . . 16
S6 Algorithmic Detection of Community Structure in State-Space Mappings . . . 17

SUMMARY

Synopsis. In the main text, we related human brain anatomical connectivity to functional brain connectivity measured at rest, during the performance of an attention task, and during the performance of a memory task. We found that resting, attention, and memory states were differentially supported by structural connectivity within and between known functional networks. The relationship between anatomy and function was used to assign a measure of separation between cognitive states, and we found that individual variability in this separation was related to variability in behavioral performance during attention and memory tasks.

Summary of Model and Results. To perform our analyses, we first identified brain regions that overlapped with the task-positive and task-negative networks identified in [1], and we refer to the remaining regions as "other" regions. From the set of task-positive, task-negative, and other regions, we constructed subject-averaged ("representative") and subject-specific brain networks whose connections were weighted by measures of structural connectivity (SC; number of white matter streamlines) and functional (FC; strength of BOLD correlation) connectivity, as shown in Figure 1 of the main text). To reliably compare representative and subject-specific networks, we restricted our analysis to the subset of region pairs that were consistently structurally connected within a large percentage (80%) of subjects. Within this subset of region pairs, we distinguished couplings between two task-positive regions (PP), two task-negative regions (NN), and one task-positive and one task-negative region (PN). These couplings were compared to the remaining set ($\{*O\}$) of couplings between one task-positive and one other region (PO), one task-negative and one other region (NO), and two other regions (OO). We quantified the number density n (defined to be the fractional number of all structural connections) linking a given type of region pair coupling. We then examined changes Δn in number density while biasing toward increasingly strong correlations above a variable threshold τ.

We found that the distributions of Δn_{PP}, Δn_{NN}, and Δn_{PN} distinguish between strong resting-, attention-, and memory-state correlations in the representative brain network. This was illustrated by constructing a state-space mapping of structure-function relationships defined by two quantities, $\Delta n_{PP} - \Delta n_{NN}$ and Δn_{PN}, that differentially vary as a function of the resting-state (τ_R), attention-state (τ_A), and memory-state (τ_M) thresholds used to select strongly-correlated region pairs (shown in Figure 2 of the main text). This state-space mapping revealed a large degree of separation between resting, attention, and memory states.

We then examined the extent to which the separations observed in the representative brain network were consistently maintained within subject-specific brain networks. Each subject was described by a triad of points given by the resting-, attention-, and memory-state distribution averages $(\langle \Delta n_{PP} - \Delta n_{NN} \rangle \ , \ \langle \Delta n_{PN} \rangle)$. The degree of separation between two cognitive states could then be quantified by the angular separation $\Delta\theta$ between two distribution averages, with smaller separations indicating that two cognitive states were supported by similar types of structural couplings. We found that subjects naturally organized into primary and secondary groups based on the relative separation between resting, attention, and memory states. The primary group showed the smallest separations between the two task states (attention and memory), while the secondary groups showed the smallest separation between rest and one task state.

The primary and secondary groupings, identified based on the the state-space mapping, showed significant differences from one another in behavioral measures of attention and memory task performance. The secondary groups of subjects, being outliers in brain architecture, were found to be outliers in attention and memory task performance. Furthermore, the secondary groups, showing larger state-space separations between attention and memory states, also show larger differences between attention versus memory performance.

Together, these results demonstrate that structural couplings within and between task-related networks differentially support strong, task-dependent functional correlations. Furthermore, the degree to which cognitive states differ from one another, as measured by such structure-function relationships, is predictive of behavior.

Summary of SI Content. In the following supporting material, we first describe the experimental methods and participants of this study. We then describe the construction of task-positive and task-negative networks, and we highlight the specific anatomical regions of the brain involved in the task-related network couplings PP, NN, PN, and $\{*O\}$. We then show the distributions of resting- (FC_R), attention- ($\Delta\mathrm{FC}_R$), and memory-state ($\Delta\mathrm{FC}_R$) functional correlations between PP, NN, and PN region pairs. By thresholding these distributions of FC, we show the resulting variations Δn_{PP}, Δn_{NN}, and Δn_{PN} in the number density of structural connections linking strongly-correlated region pairs. Together, these distributions of Δn were used to define the state-space mapping of resting, attention, and memory states shown in Figure 2 of the main text. We show the observed

separation between cognitive states is robust to the specific choices made in constructing the representative brain network and in thresholding the resulting distributions of FC.

When compared across subjects, this state-space mapping revealed significant inter-subject organization in the relative separations between cognitive states, with subjects naturally organizing into primary and secondary groups (Figure 3 of the main text). We show that the observed organization into such groups is not an artifact of our specific analysis techniques, and we confirm that the primary and secondary groups are statistically similar to the groups identified by a clustering algorithm. Lastly, these groups were shown in Figure 4 of the main text to exhibit significant differences in behavioral task performance. Here, we show the full set of ANOVA results that were used to confirm the statistical significance of these performance differences.

MATERIALS AND METHODS

Structural and functional brain scans were performed at the UCSB Brain Imaging Center. Functional brain scans were separately acquired while subjects were at rest, while performing an attention task, and while performing a facial recognition memory task. Behavioral data was collected during both the attention and memory tasks. Specific details regarding experimental protocol and estimation of structural and functional connectivity can be found in [2, 3]. Details regarding the assessment of behavioral task performance can be found in [3]. We analyzed data from 71 subjects: ages 27-45 (mean 34.51, standard deviation 4.28); 1 female, 71 males; all right-handed. None of the subjects were color blind. Informed written consent was obtained from each subject prior to the experimental sessions. All procedures were approved by the University of California, Santa Barbara Human Subjects Committee.

Attention and Memory Task Design

Functional scans were acquired during three different experimental states: resting, attention, and memory for faces. The resting-state scan was acquired while subjects were asked to fixate on a blank screen. Task scans were acquired while subjects performed strategic attention and memory tasks, as described below. For additional details regarding task design, see [3] and the SI Appendix of [2].

Attention Task

The strategic attention task consisted of a brief unscanned practice session followed by a scanned test session. The task employed a modified spatial cueing procedure [4] in which subjects were asked to view sequences of visual stimuli, presented in blocks of discrete experimental trials, and discriminate test displays for the presence or absence of a pre-specified target stimulus. Each trial consisted of a 250ms presentation of a blank screen, followed by the sequential presentation of a fixation cross ('+'), an arrowhead cue stimulus, and a test display in which a target could be present or absent. The fixation cross appeared at the center of the viewing screen for a randomly selected duration between 400ms and 600ms and was subsequently replaced by an arrowhead cue pointing to the left or to the right of center. The color of the arrowhead (either red or green) indicated the likelihood that the target would be present in the subsequent test display, such that one arrow color indicated a 70% likelihood that the target would appear, while the other color indicated 30% likelihood. The direction of the arrowhead indicated the probable (75%) location of the target should it appear at all.

A test display was presented within a random time interval (between 1400ms and 160ms) following the cue onset. Two small rectangles, one on each side of the cue, appeared for approximately 50ms. Horizontal rectangles were defined as targets, while vertical rectangles were defined as distractors. Each test display contained either one target stimulus and one distractor (target present condition), or two distractors (target absent condition). The test display was replaced by a central fixation cross for an inter-trial interval that varied between 1200ms and 3200ms. Following each test display, subjects were required to make a two-alternative forced choice button press response to indicate whether or not a target had been present.

Memory Tasks

The memory task consisted of a unscanned study session followed by a scanned test session. During the test session, subjects were asked to view black-and-white photographs of faces and determine whether or not the face had been previously presented during the study session.

360 black-and-white photographs of faces of varying ethnicity were used as stimuli. During the study session, 180 stimuli were presented for a duration of 1000ms, followed by an inter-trial interval of 1000ms. The study session was followed by the test session, in which 180 previously-studied stimuli and 180 novel stimuli were presented. Test stimuli were divided into two conditions: a high probability condition in which there a 70% probability that the stimulus was old (i.e., studied previously), and a low probability condition in which there was a 30% probability that the stimulus was old. Probability condition was cued via a colored rectangular frame (red or green). Stimuli were presented in a pseudo-block format in which six to nine trials of the same probability were presented before the probability switched. Old and new stimuli were intermixed within these blocks. The probability indicated by the color cue corresponded to the distribution of old and new stimuli within the overall test session rather than the distribution within a given pseudo-block. A set of 180 fixation trials (in which a central fixation cross was displayed for 2500ms) was intermixed throughout the entire test session. Following each test display, subjects were required to make a two-alternative forced choice button press response to indicate whether or not the stimulus had been previously studied.

Connectivity Estimates

Structural Connectivity

Structural connectivity is estimated from diffusion tensor imaging (DTI) scans using a deterministic streamline tractography algorithm. Weighted connectivity measures estimated from DTI data are highly reproducible across subjects, with high intra-class correlation coefficients (ICC$>$ .72) and low coefficients of variation (CV$\sim$ 4%) [5]. However, the deterministic algorithm used here can fail to distinguish branching or crossing fibers, and they can similarly fail to identify fiber pathways that exhibit abrupt turns [6]. Connectivity estimates might be improved through the use of probabilistic, rather than deterministic, tractography algorithms (e.g. [7]). Furthermore, it is possible that the number of long fibers is underestimated in our data, as currently tractography algorithms are biased toward detecting short fiber pathways.

Functional Connectivity

We have examined functional connectivity while subjects are at rest and while performing attention- and memory-demanding cognitive tasks. This work employs one possible approach for characterizing brain dynamics by functional connectivity strength. Alternative approaches, such as psychophysiological interactions (PPI), can be used to characterize brain dynamics by the level of activity within brain regions [8, 9]. While recent fMRI studies suggest that resting-state activity and connectivity might be correlated with one another [10, 11, 12], the relationship between activity and connectivity across task-driven states remains unknown.

The regions constructed from the AAL atlas consisted predominantly of gray matter. However, these regions may have contained some voxels of cerebrospinal fluid (CSF) or white matter. Signal variance in these two tissues, which is related to physiological noise such as heart rate and respiration, may have contributed to the strength of correlation between different regions. However, the fraction of white matter and CSF is relatively small, and any related contribution to inter-regional correlation strength would likely be constant across cognitive states.

RESULTS

Brain Regions Involved in Network Couplings

In the main manuscript, we assessed the strengths of structural and functional couplings between 600 cortical and subcortical brain regions. This "uniform-600 atlas," upsampled from the anatomical AAL atlas [13], was constructed to create a set of roughly equally-sized regions that obey gross anatomical boundaries and has been previously used to assess human brain structural and functional connectivity [2].

Using this uniform-600 atlas, we isolated couplings (both structural and functional) within and between the task-positive and task-negative networks identified in [1]. In order to identify the regions within the uniform-600 atlas that participate in these task-positive and task-negative networks, we first performed a labeling of the anatomical AAL atlas into "task-positive," "task-negative," and "other" regions. We then identified the corresponding "task-positive," "task-negative," and "other" regions in the upsampled uniform-600 atlas. This procedure, of first labeling the AAL atlas and then upsampling labeled regions to construct the uniform-600 atlas, minimizes the frequency of boundary regions and more closely preserves the number balance between task-positive and task-negative regions originally used in [1] (further discussed in the following paragraph).

Labeling of the Anatomical AAL Atlas

The labeling of the anatomical AAL atlas was performed as follows: 29 voxels (16 "task-positive" and 13 "task-negative" voxels) were originally identified in [1], from which we created 29 spherical regions of interest (ROIs). ROIs were chosen to be 5mm in radius, constructed in Talairach coordinates and converted to MNI coordinates. Anatomical regions that overlapped with either a task-positive or a task-negative ROI were respectively labeled "task-positive" or "task-negative." Anatomical regions that did not overlap with either a task-positive or a task-negative ROIs were labeled as "other" regions. For anatomical regions that overlapped with both a task-positive or task-negative ROI, the anatomical region was labeled based on a winner-takes-all strategy according to the task-positive or task-negative ROI with which the anatomical region had greater overlap (e.g. an anatomical region with 50% of its volume overlapping with a task-positive ROI and 30% of its volume overlapping with a task-negative ROI was labeled as "task-positive"). Of the 90 regions in the AAL atlas, only 3 were boundary regions assigned using this winner-takes-all strategy. Table S1 enumerates the region label assignments of all "task-positive," "task-negative," and "other" anatomical regions in the AAL atlas.

Using these region label assignments, we were able to differentiate between the sets of couplings (both structural and functional) that link two task-positive regions (PP), two task-negative regions (NN), and a task-positive and task-negative region (PN) from the set of couplings $\{*O\}$ that link an "other" region with one task-positive region (PO), one task-negative region (NO), or a second "other" region (OO). We restricted our analysis to the sets of region pairs that were consistently structurally connected within 80% of subjects. Figure S1 shows the anatomical regions in the AAL atlas linked by these consistent structural couplings, where couplings are color-coded by the type of regions (task-positive, task-negative, and other) that they link. Alternating rows and columns indicate the same anatomical regions in the right versus left hemispheres, and highlighted squares indicate that the corresponding anatomical regions are involved in a given type of PP, NN, PN, or $\{*O\}$ coupling. It is important to note that the brain regions involved in the task-positive and task-negative networks were originally defined based on functional (but not structural) measures [1]. Here, we consider only those task-positive and task-negative region pairs that are structurally connected within a large percentage of subjects.

We find that the majority of structural couplings shown in Figure S1 lie close to the diagonal, indicating that these structural couplings link two regions within a given anatomical lobe. The majority of PP couplings link two regions within the frontal, occipital, or parietal lobes, while the majority of NN couplings link two regions within the frontal and limbic lobes. PN couplings link regions within and between frontal, occipital, parietal, and limbic lobes. Off-diagonal elements in Figure S1 indicate couplings that link regions within different lobes and are observed between frontal and central regions (PP), between limbic, occipital, and parietal regions (NN), and between these and subcortical and temporal regions ($\{*O\}$).

Upsampling to the Uniform-600 Atlas

Given that each region in the uniform-600 atlas is fully contained within a specific anatomical region in the AAL atlas, we used anatomical regions in the AAL atlas to label the corresponding "task-positive", "task-negative", and "other" regions within the upsampled uniform-600 atlas. This upsampling procedure is advantageous because it allows us to assess structural and functional connectivity between regions that are roughly equal in size, and it allows us to compare our findings to previous analyses of structural and functional human brain connectivity [2]. In a manner analogous to that performed with the anatomical AAL atlas, we then identified the structural and functional couplings that link PP, NN, PN, and $\{*O\}$ region pairs within the uniform-600 atlas. All analyses presented in the main manuscript, and all subsequent analyses discussed in the SI Appendix, were performed using the set of consistently structurally-connected regions pairs (region pairs linked by anatomical connections within 80% of subjects) within the labeled uniform-600 atlas.

Contributions of Network Couplings to Strong FC

The analysis described in the main text assessed changes in number density Δn as a function of increasingly strong FC. To perform this analysis, a variable threshold τ was used to select strongly-correlated region pairs with FC$> \tau$. Within this subset of region pairs, we calculated the number density n of structural connections (measured as a fraction of all structural connections) linking two task-positive regions (n_{PP}), two task-negative regions (n_{NN}), and a task-positive and task-negative region (n_{PN}). Changes Δn in number densities were then computed relative to the baseline number density of PP, NN, and PN connections measured in the absence of functional thresholds.

Table S1: ROI indices in anatomical AAL atlas assigned labels "task-positive," "task-negative," and "other." The total number of regions in each category are given for both the AAL atlas (denoted AAL90) and the uniform-600 atlas (denoted U600).

Region Labels	ROI Indices in AAL90	# in AAL90	# in U600
Task-Positive	1, 2, 4, 7, 8, 11-13, 17-20, 30, 50-52, 54, 57-61, 86, 89	24	240
Task-Negative	3, 23-26, 28, 33, 34, 39, 40, 43, 55, 56, 66, 67, 90	16	128
Other	5, 6, 9, 10, 14-16, 21, 22, 27, 29, 31, 32, 35-38, 41-44 45-49, 53, 62-65, 68-85, 87, 88	50	232

The process of extracting the number density of connections linking strongly correlated region pairs was illustrated in Figure 1c in the main text, for which the threshold τ_R was imposed on the distribution of resting-state FC, and the quantities Δn_{PP}, Δn_{NN}, and Δn_{PN} were computed for each value of τ_R. Although not illustrated in the same subfigure, analogous techniques were applied to the attention- and memory-state distributions of FC. Here, we show the full distributions of resting-state (FC_R), attention-state (FC_A), and memory-state (FC_M) functional connectivity that were respectively thresholded with τ_R, τ_A, and τ_M (Figure S2a-c). We then show the resulting distributions of Δn_{PP}, Δn_{NN}, and Δn_{PN} computed across variations in thresholds τ_R, τ_A, and τ_M (Figure S2d-f).

In the resting state, both PP and NN region pairs are more strongly correlated than PN region pairs (Figure S2a), a finding that agrees with the original definition of task-positive and task-negative networks based on strong correlations within networks (represented here as strong PP and NN correlations) and anticorrelations between networks (represented here as weak PN correlations). In the attention state, PP region pairs are more strongly correlated, while NN region pairs are more weakly correlated (Figure S2b), a finding that further supports the known increase versus decreases in activity of task-positive versus task-negative regions during attention tasks relative to rest [1]. In the memory state, PN region pairs are more strongly correlated than either PP or NN region pairs (Figure S2c).

We find that the resulting distributions of Δn, calculated from the thresholded distributions of FC, vary significantly between resting, attention, and memory states (Figure S2d-f). In the resting state, we see increased contributions from NN couplings and decreased contributions from PP connections to strong FC_R (Figure S2d). We see the opposite trend in the attention state, with increased contributions from PP couplings and decreased contribution from NN couplings to strong FC_A (Figure S2e). The memory state differs from both attention and rest, showing increased contributions from PN connections to strong FC_M (Figure S2f).

Together, the distributions of Δn shown in (Figure S2d-f) were used to construct the state-space mapping discussed in the main text. For each cognitive state, the quantities $\Delta n_{PP} - \Delta n_{NN}$ and Δn_{PN} were computed across functional thresholds τ_R, τ_A, and τ_M, respectively producing the resting-, attention-, and memory-state distributions shown in Figure 2 of the main text.

Robustness to Analysis Variations

Thresholding

In constructing the representative brain network described in the main text, we selected the subset of region pairs that were consistently structurally connected within 80% of subjects. We then examined the changes in the density of within-network ($\Delta n_{PP} - \Delta n_{NN}$) versus between-network (Δn_{PN}) couplings as we biased toward strong FC by increasing the functional threshold τ. Because this thresholding process incrementally restricts the number of region pairs in consideration, large values of τ can be accompanied by increased jitter in the distributions of $\Delta n_{PP} - \Delta n_{NN}$ and Δn_{PN}. In the main manuscript, we imposed an upper bound on τ in order to remove this jitter. We now show that the results shown in the main text are robust to variations in inter-subject consistency in SC and in the bounds imposed on the functional threshold τ.

To demonstrate this robustness, we repeat the analysis described in the main text by comparing the density of within- versus between-network couplings as a function of the increasing thresholds τ_R (resting state), τ_A (attention state), and τ_M (memory state). The resulting state-space mapping was shown in Figure 2 of the main text and revealed a large degree of separation between resting, attention, and memory states. To evaluate the

robustness of this state-space across analysis variations, we compute the distribution averages $\langle\Delta n_{PP}-\Delta n_{NN}\rangle$ and $\langle\Delta n_{PN}\rangle$ as a function of inter-subject consistency in SC (Figure S3a) and as a function of the bounds on τ (Figure S3b). We show that, in both analysis variants, the observed separation between brain states is consistently observed across threshold values.

When increasing the inter-subject consistency in SC, both the resting- and attention-state distributions move further from the origin, while the memory-state distribution moves closer to the origin (Figure S3a). In agreement with the results of the main text, this finding suggests that increasingly robust structural connectivity facilitates an increasingly strong separation between rest and attention states, with memory sharing features of both.

When varying the bounds imposed on τ, we consider two cases in which we either increase the minimum bound on τ or decrease the maximum bound. Because our methods examine changes Δn between region pairs with FC $> \tau$, the largest values of Δn (and therefore the largest separations between states) are observed in the large-τ tails of the distributions. As a result, increasing the minimum bound and decreasing the maximum bound on τ have opposite effects (Figure S3b). By increasing the minimum bound on τ, we see an increasingly large separation between all states. Conversely, when decreasing the maximum bound on τ, we see a decrease in the separation between all states.

Weighted versus Unweighted Network Representations

In the main manuscript, we evaluated changes in the patterns of structural connectivity linking PP, NN, and PN region pairs as we incrementally biased toward strong FC. This procedure employed a complex network representation in which brain regions are represented as nodes in a complex network. A given pair of nodes is then defined to be connected if it is linked by one or more structural connections within 80% of subjects, and unconnected otherwise.

In the main text, we considered a weighted network representation in which connections were weighted by the number of white matter tracts linking two regions. We then measured changes $\Delta n(\tau)$ in the fractional number density of connections linking task-positive and task-negative networks as we biased toward strongly-correlated region pairs above a sliding threshold value τ. We compared two quantities: the change in PN density (Δn_{PN}) and the relative changes in PP versus NN densities ($\Delta n_{PP}-\Delta n_{NN}$). These results were shown in Figure 2 of the main text and are shown again here in Figure S4a across variations in the functional thresholds τ_R (resting state), τ_A (attention state), and τ_M (memory state).

Consistent results are observed if we instead consider an *unweighted* network representation for which we measure changes in the fractional number of strongly-correlated region pairs, rather than the fractional number of structural connections linking these region pairs. Figure S4b compares changes in the fractional number of strongly correlated PP, NN, and PN region pairs as a function of τ_R, τ_A, and τ_M. Consistent with the results shown in the main text, we find that changes in the fractional number of strongly correlated region pairs distinguish between resting, attention, and memory states (Figure S4a). The observed separation, however, is more pronounced in the weighted as compared to the unweighted network (Figure S4b), suggesting that structural information plays an important role in supporting strong, task-dependent functional correlations.

Comparisons of State-Space Mappings with Null Model

The state-space mapping of structure-function relationships shown in Figure 2 of the main text, which compares changes in within-network ($\Delta n_{PP}-\Delta n_{NN}$) versus between-network (Δn_{PN}) couplings, revealed a large degree of separation between resting, attention, and memory states in the representative brain network. To investigate whether this separation was consistently maintained within individual subjects, we constructed an analogous state-space mapping for each individual subject. To compare across subjects, we compactly represented each subject by a triad of points describing the resting (R), attention (A), and memory-state (M) distribution averages $\langle\Delta n_{PN}\rangle$ and $\langle\Delta n_{PP}-\Delta n_{NN}\rangle$ (Figure S5a and Figure 3a of the main text). To isolate inter-subject variability in the separation between states, we performed a remapping in which each subject was described by a triangle whose vertices were given by cyclical permutations of $(\Delta\theta_{ab},\Delta\theta_{bc})$, where $\Delta\theta_{ab}$ describes the angular separation between states a and $b\in\{R,A,M\}$ (Figure S5b and Figure 3b of the main text). We found that this remapping identified patterns of organization across subjects, with a majority of subjects showing small separations between attention and memory states (Figure S5c-d and Figure 3d of the main text). These patterns of organization naturally isolated groups of subjects with similar structure-function relationships, and we found that similarities in such structure-function relationships were linked to similarities in behavioral task performance.

To confirm that the observed patterns of organization are not an artifact of our analysis techniques, we compare our results to those obtained from a null model in which "task-positive," "task-negative," and "other" region labels are randomly reassigned to regions within our uniform-600 atlas. This comparison, shown in Figure S5c-f, reveals that random label assignment removes the observed patterns of organization across subjects. In the original data, a large percentage of subjects showed similar separations between resting, attention, and memory states. This is illustrated in Figure S5d by clusters of markers of the same type and by overlapping triangles linking these markers. Under random label reassignment, illustrated in Figure S5f, we no longer see clustering of markers of the same type, nor do we see overlapping sets of triangles linking these markers. If we additionally assess behavioral measures of subjects grouped based on the null-model state-space, we no longer see significant differences in performance between groups of subjects with different structure-function relationships (described in detail in the following sections). Together, these results confirm that the patterns of organization observed in the original data, which naturally isolate groups of subjects with similar structure-function relationships, is not an artifact of our specific analysis techniques.

Algorithmic Detection of Community Structure in State-Space Mappings

The subject-specific state-space mappings, shown in Figure S4 and in Figure 3 of the main text, naturally isolated one primary and two secondary groups of subjects who showed similarities in the relative separation between brain states. The primary group (66% of subjects) showed the smallest separation between attention and memory states, while the two secondary groups showed the smallest separation between rest and memory (23% of subjects) and between rest and attention (11% of subjects).

We now confirm that the groups identified by this state-space representation are statistically similar to groups identified algorithmically using community detection techniques [14, 15]. As shown in Figure 3a of the main manuscript, each subject can be compactly represented by a triad of resting- (R), attention- (A), and memory-state (M) distribution averages of Δn_{PN} and $\Delta n_{PP} - \Delta n_{NN}$. For each subject-specific triad, we quantify the angular position θ of individual distribution averages and the angular separation $\Delta\theta$ between pairs of distributions averages, such that a given subject i can be described by a vector $v_i = [\theta_R; \theta_A; \theta_M; \theta_{RA}; \theta_{AM}; \theta_{MR}]$. We estimate the similarity between each pair of subjects i and j in state-space by computing the absolute value of the Pearson correlation coefficient between v_i and v_j to produce the unsigned $J \times J$ similarity matrix $\mathbf{S}$ shown in Figure S6a.

To identify separate groups of subjects with similar state-space structure, we treat the similarity matrix $\mathbf{S}$ as a weighted undirected network in which J nodes (subjects) are connected by E edges (similarities between pairs of subjects in their state-space structure). We then use a quality function Q to measure the quality of a partition of subjects into groups or communities. Here, we define the quality function using the popular choice of *modularity* ([17, 18, 19]):

$$Q = \sum_{ij} [S_{ij} - \gamma W_{ij}] \delta(g_i, g_j) \,, \tag{0.1}$$

where node i is assigned to community g_i, node j is assigned to community g_j, the Kronecker delta $\delta(g_i, g_j) = 1$ if $g_i = g_j$ and it equals 0 otherwise, γ is a resolution parameter (which can be used to tune the mean size of communities), and W_{ij} is the expected weight of the edge connecting node i to node j under a specified null model. We employ the common choice $\gamma = 1$, but note that other values of γ can be used to examine groups of different sizes [20, 14, 21]. We also employ the most common modularity-optimization null model used in undirected, single-layer networks: the Newman-Girvan null model $W_{ij} = \frac{k_i k_j}{2m}$ [14, 15, 17, 18, 19], where $k_i = \sum_j S_{ij}$ is the strength of node i, and $m = \frac{1}{2} \sum_{ij} S_{ij}$. Maximization of Q yields a hard partition of a network into communities such that the total edge weight inside of communities is as large as possible (relative to the null model and subject to the limitations of the employed computational heuristics, as optimizing Q is NP-hard [14, 15, 22]). In the present study, this procedure enables us to identify groups of subjects with similar state-space structure.

Because the modularity quality function typically has a large number of nearly-degenerate local optima for networks constructed using real data, we optimize the modularity quality function 100 times to create the set of partitions shown in Figure S6b. We then extract a representative, or *consensus*, partition from the set of partitions using a consensus partition method that employs a null model to correct for statistical noise in sets of partitions [16]; see Figure S6c. This consensus partition identifies 3 communities of subjects with similar state-space structure (see Figure S6d). The largest community contained 30 subjects, the second largest community contained 24 subjects, and the smallest community contained 17 subjects.

To determine whether the consensus partition obtained in the algorithmic detection of community structure was statistically similar to the state-space partitions defined in the main manuscript, we employ the z-score of the Rand coefficient as a measurement of partition similarity [23]. For comparing two partitions α and β, we calculate the Rand z-score in terms of the network's total number of pairs of nodes L, the number of pairs L_α that are in the same community in partition α, the number of pairs L_β that are in the same community in partition β, and the number of pairs $w_{\alpha\beta}$ that are assigned to the same community both in partition α and in partition β. The z-score of the Rand coefficient comparing these two partitions is given by:

$$z_{\alpha\beta} = \frac{1}{\sigma_{w_{\alpha\beta}}} \left(w_{\alpha\beta} - \frac{L_\alpha L_\beta}{L} \right) , \tag{0.2}$$

where $\sigma_{w_{\alpha\beta}}$ is the standard deviation of $w_{\alpha\beta}$ (as in [23]).

We find that the consensus partition obtained from the algorithmic detection of community structure in the state-space similarity matrix is statistically similar to the partition identified in the main text based on groupings of state-space relationships; the associated z-score of the Rand coefficient was 12.1, which has an associated two-tailed p-value of less than 0.0001. These results confirm that the relative separation between cognitive states, used in the main manuscript to partition groups of subjects , captures the purely data-driven organization of subject-specific structure-function relationships.

ANOVA Results

The primary and secondary groups of subjects, identified from the state-space mapping based on the relative separation between cognitive states, showed significant differences in behavioral performance during both attention and memory tasks. The significance of these behavioral differences was confirmed using a repeated measures analysis of variance (ANOVA) implemented via the MATLAB routine "Mixed Between/Within Anova" (http://www.mathworks.com/matlabcentral/fileexchange/27080-mixed-betweenwithin-subjects-anova/). Here, we give the full ANOVA results using the original data, and we compare the results to those obtained using the null model in which "task-positive," "task-negative," and "other" region labels are randomly reassigned.

We examined three performance measures: reaction time (RT) between stimulus presentation and response; criterion switch score (CS), a measure of strategic flexibility in decision making strategies; and d-prime (d'), a measure of sensitivity as related to accuracy. A more detailed discussion of these performance measures can be found in the main manuscript and in [3]. Performance measures were assessed during both attention (A) and memory (M) tasks, for a total of six behavioral measures (three each for attention and memory). We then measured both absolute ($|q_A - \bar{q}_A|$, $|q_M - \bar{q}_M|$) and relative ($|q_A - q_M|/(q_A + q_M)$) differences in a given performance measure q, where $\bar{q}$ denotes the group average computed across subjects. A repeated measures ANOVA was performed using a total of nine behavioral variables as repeated measures: $|q_A - \bar{q}_A|$, $|q_M - \bar{q}_M|$, and $|q_A - q_M|/(q_A + q_M)$, where q takes values of RT, CS, and d'. Using the three groupings identified from the state-space mapping, we distinguished between the primary group (subjects for whom attention and memory states show the smallest separations) and the two secondary groups (subjects for whom either rest and attention states or rest and memory states show the smallest separations). Our categorical measures were then taken to have one of two values to distinguish between the primary versus secondary groupings identified from the state-space mapping.

The results of the repeated measures ANOVA are highlighted in Tables S2 and S3 using both the original data and the null model. Using the original data, the primary and secondary groups differed significantly from one another, with a main effect of grouping of $F(1) = 4.51$ and $p = 0.037$. These results confirm that subjects who show differences in structure-function relationships also show statistically significant differences in behavioral performance. In comparison, the primary and secondary groups identified in the null model did not differ significantly from one another, with a main effect of grouping of $F(1) = 1.68$ and $p = 0.20$. The null model finding that random label-reassignment removes the observed correlation between brain state and behavior further confirms that the statistical significance of these results is not an artifact of our analysis techniques.

Table S2: Repeated measures ANOVA results using original data. The results include the sum of squares (SSq), degrees of freedom (df), mean squares (MSq), F-statistics (F), and P-values (p). State-space groupings of subjects show significant differences in behavioral performance, with a main effect of grouping of $F(1) = 4.51$ and $p = 0.037$.

**Source**	**SSq**	**df**	**MSq**	**F**	**p**
Between-subjects factor	8126.141	1	8126.141	4.514	0.0372070
Between subjects error	124219.473	69	1800.282		
Within-subjects factor	20068.502	8	2508.563	0.713	0.6797666
Within-subjects error	1940842.301	552	3516.019		
Within- × between-subjects interaction	13085.645	8	4917.354	1.399	0.1940794

Table S3: Repeated measures ANOVA results using a null model in which "task-positive," "task-negative," and "other" region labels are randomly reassigned. The results include the sum of squares (SSq), degrees of freedom (df), mean squares (MSq), F-statistics (F), and P-values (p). State-space groupings of subjects in the null model do not show significant differences in behavioral performance, with a main effect of grouping of $F(1) = 1.68$ and $p = 0.20$.

**Source**	**SSq**	**df**	**MSq**	**F**	**p**
Between-subjects factor	3143.414	1	3143.414	1.679	0.1994110
Between subjects error	129202.199	69	1872.496		
Within-subjects factor	10782.821	8	1347.853	0.379	0.9320364
Within-subjects error	1965337.264	552	3560.394		
Within- × between-subjects interaction	14843.871	8	1855.484	0.521	0.8409027

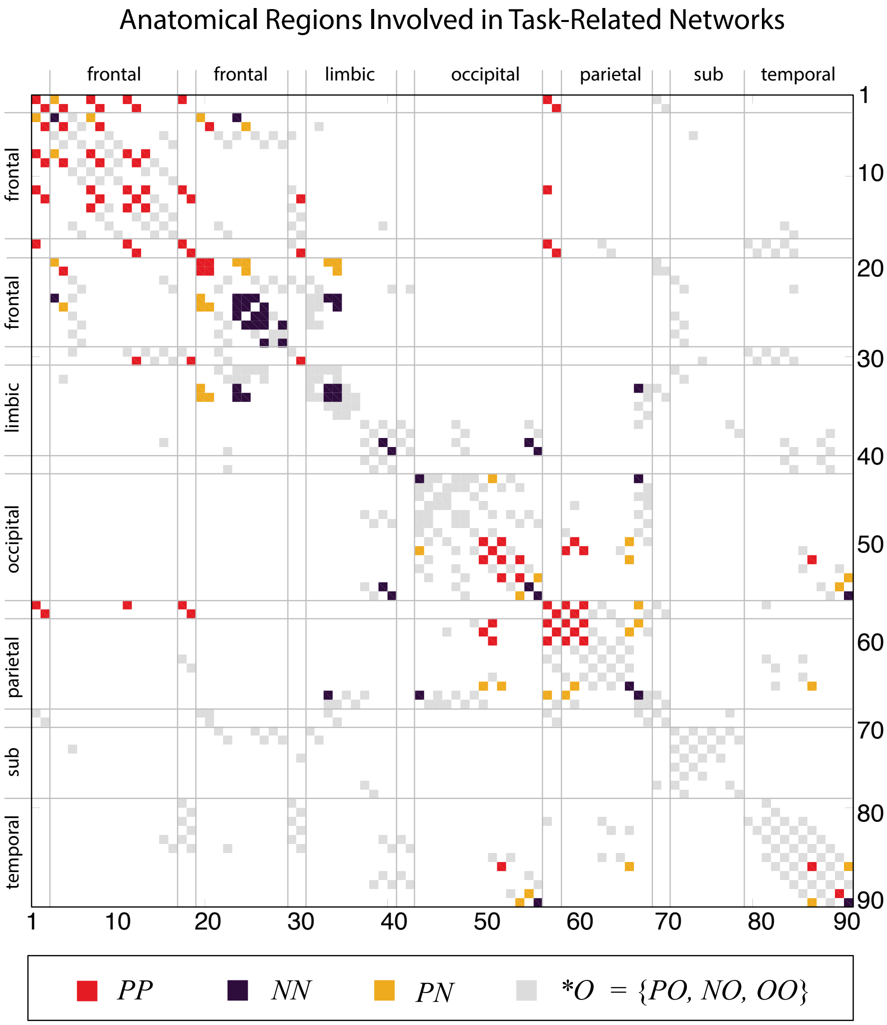

Figure S1: **Brain Regions Involved in Network Couplings.** Regions are numbered according to their label in the 90-region AAL atlas, with odd and even numbers respectively indicating regions within left and right hemispheres. Horizontal and vertical lines delineate the following anatomical groupings: frontal lobe, limbic lobe, occipital lobe, parietal lobe, subcortical regions, and temporal lobe. Unlabeled regions between groupings indicate central regions, including the pre/postcentral gyri and rolandic operculum. Colored squares mark structurally-connected region pairs in the representative brain network, with different colors distinguishing between PP (red), NN (purple), and PN (yellow), and $\{*O\}$ (gray) couplings. PP couplings tend to involve frontal, parietal, and occipital regions, while NN couplings tend to involve frontal and limbic regions. PN and $\{*O\}$ couplings can be found linking regions within each of these anatomical groupings. The majority of off-diagonal components, which link regions from different anatomical groupings, involve subcortical regions and regions from the temporal lobe.

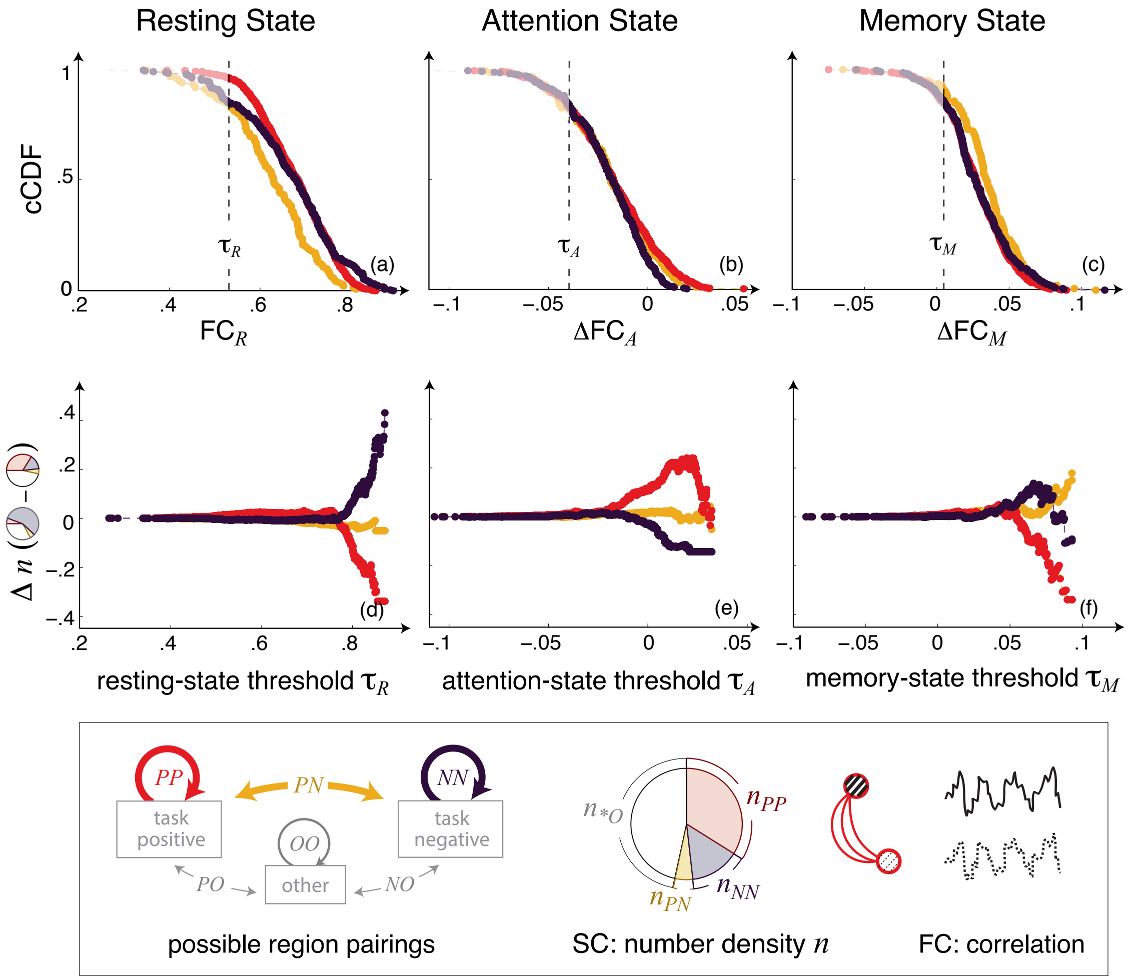

Figure S2: **Contributions of Network Couplings to Strong Functional Connectivity** We consider couplings between two task-positive regions (PP), two task-negative regions (NN), and one task-positive and one task-negative region (PN). Top row: Complementary cumulative distribution functions (cCDFs) of functional measures FC$_R$, ΔFC$_A$, and ΔFC$_M$ produced by PP, NN, and PN connections in the representative brain network. In the resting state, PP and NN connections show slightly stronger correlations than do PN connections. In the attention state, all distributions are strongly overlapping, and in the memory state, PN connections show slightly stronger correlations than do PP and NN connections. Bottom row: We compute the total number density n of streamlines linking PP, and NN, and PN region pairs, and we evaluate changes Δn in this number density as we bias toward increasingly strong correlation above the thresholds τ (shown as vertical dotted lines in upper row). We find that strong resting-state correlations are supported by an increased contribution from NN connections but decreased contributions from PP and PN connections. Strong changes in attention-state correlations are supported by an increased contribution from PP connections but a decreased contribution from NN connections. Strong changes in memory-state correlations share similarities with both rest and attention, being supported by an increased contribution from PN and NN connections and a decreased contribution from PP connections.

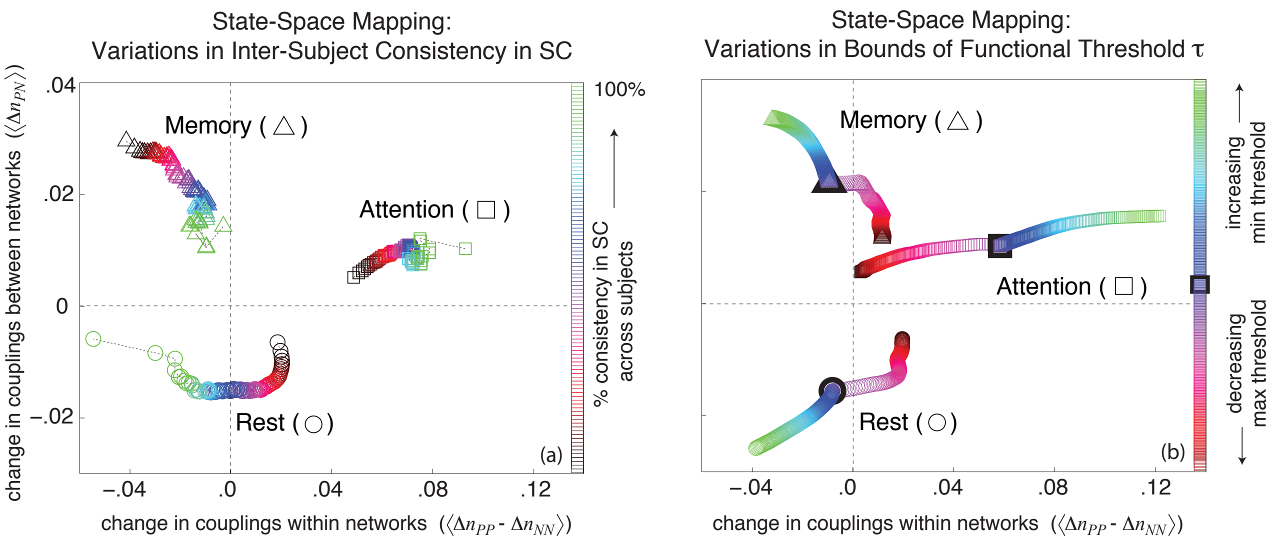

Figure S3: **Robustness of State-Space Mapping to Thresholding.** Distribution averages $\langle \Delta n_{PN} \rangle$ and $\langle \Delta n_{PP} - \Delta n_{NN} \rangle$ produced by resting (circular markers), attention (square markers), and memory (triangular markers) states as a function of (a) inter-subject consistency in SC and (b) bounds on the functional threshold τ. (a) Variations in inter-subject consistency in SC are denoted by the colorscale in subfigure (a), with black indicating that all structural connections were used in the analysis, and with light green indicating that only those connections present in 100% of subjects were used in the analysis. Across all variations in inter-subject consistency in SC, we find a strong degree of separation between different states. In biasing toward high consistency, the resting and attention states show larger deviations from the origin and greater separation from one another. In comparison, the memory state shows smaller deviations from the origin and smaller separations from the resting and attention states when biasing toward high consistency. (b) Variations in the bounds imposed on the functional threshold τ are denoted by the colorscale in subfigure (b), with the black square marker indicating the case in which no bounds were imposed on τ. Increases in the minimum bound on τ are indicated in red hues, while decreases in the maximum value of τ are indicated in green hues. The minimum (maximum) bound on τ was increased (decreased) to a maximum (minimum) value τ^*, where τ^* marks the threshold for which half of all connections have FC$>\tau^*$. Increasing the minimum value of τ increases the separation between all states, while decreasing the maximum value of τ decreases the separation. If the maximum bound becomes sufficiently small (dark red), the separation between states is removed.

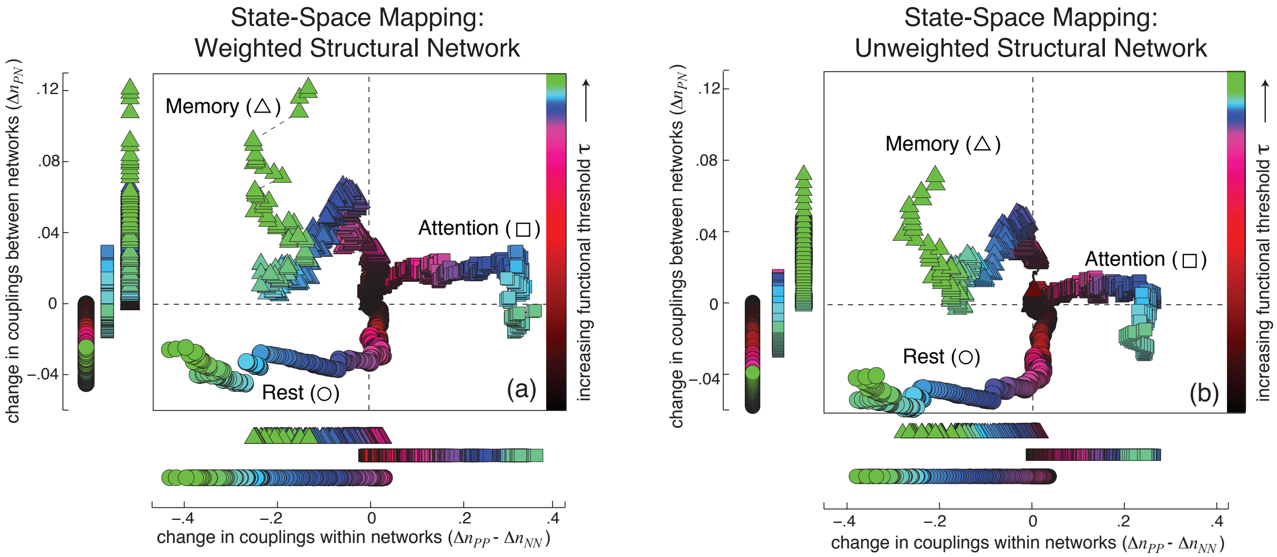

Figure S4: **Robustness of State-Space Mapping to Weighted versus Unweighted Network Representations.** State-space mapping for (a) weighted structural networks (reproduced from Figure 2 of the main text) and (b) unweighted structural networks. In both cases, the density of between-network couplings (Δn_{PN}) versus within-network couplings ($\Delta n_{PP} - \Delta n_{NN}$) is shown as a function of the increasing functional threshold τ in the representative brain network for the resting (circular markers), attention (square markers), and memory (triangular markers) states. In (a), density is computed as the fractional number of fiber tracts linking strongly-correlated regions pairs, while in (b), density is computed as the fractional number of strongly-correlated region pairs. While both weighted and unweighted networks exhibit a large degree of separation between rest, attention, and memory states, the separation is more pronounced in the weighted network (a) than in the unweighted network (b).

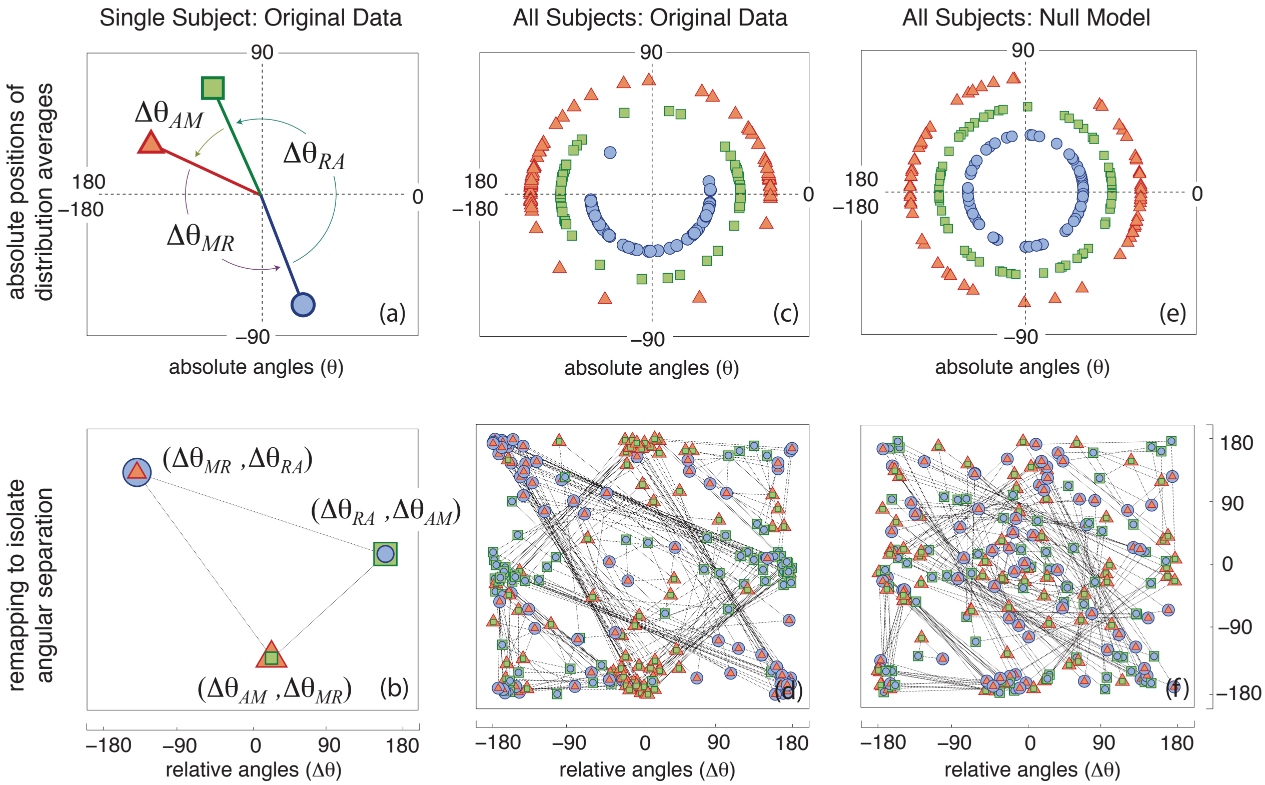

Figure S5: **Comparison of Individual State-Space Relationships to Null Model.** Comparison of original data with a null model in which "task-positive," "task-negative," and "other" region labels are randomly reassigned. (a) Each subject is compactly represented by a triad of points marking the resting-, attention-, and memory-state distribution averages of $\Delta n_{PP} - \Delta n_{NN}$ and Δn_{PN}. (b) Each subject specific triad is remapped to a triangle whose vertices are given by cyclical permutations of the angular separation $\Delta\theta_{ab}, \Delta\theta_{bc}$ between states. (c, d) In the original data, subjects naturally form groups that can be distinguished by the relative separation between resting, attention, and memory states. These groups can be identified visually by the large clustering of markers of the same type, and similarly by the large degree of overlap between triangles linking these clusters. (e,f) In the null model, the observed organization across subjects is removed; there is no clustering of markers of the same type, nor are there sets of overlapping triangles linking clusters of markers.

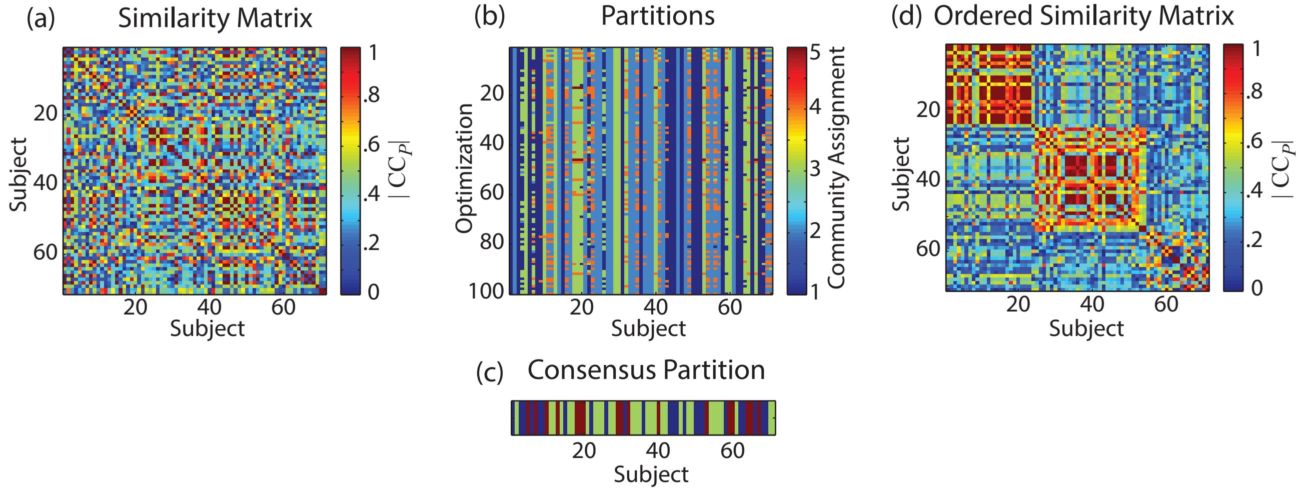

Figure S6: **Algorithmic Detection of Community Structure in State-Space Mappings.** (a) The similarity matrix **S** whose elements S_{ij} give the absolute value of the Pearson correlation coefficient ($|CC_P|$) between the state-space variable vector of subject i and of subject j. (b) The set of 100 partitions of subjects into communities obtained from optimizing the modularity quality function. Color indicates community assignment. (c) The consensus partition of subjects into communities obtained from the set of partitions in panel (b) using a method that corrects for statistical noise in sets of partitions by comparing to a null model [16]. Color indicates community assignment in the consensus partition (d) The similarity matrix **S** reordered according to community assignment in the consensus partition.

REFERENCES

[1] M. D. Fox, A. Z. Snyder, J. L. Vincent, M. Corbetta, D. C. V. Essen, and M. E. Raichle, "The human brain is intrinsically organized into dynamic, anticorrelated functional networks," *Proc. Natl. Acad. Sci. USA.*, vol. 102, no. 27, pp. 9673–9678, 2005.

[2] A. M. Hermundstad, D. S. Bassett, K. S. Brown, E. M. Aminoff, S. Freeman, D. Clewett, C. M. Tipper, A. Frithsen, A. Johnson, M. B. Miller, S. T. Grafton, and J. M. Carlson, "Structural foundations of resting-state and task-based neural activity in the human brain," *Proc. Natl. Acad. Sci. USA*, 2013.

[3] E. M. Aminoff, D. Clewett, S. Freeman, A. Frithsen, C. Tipper, A. Johnson, S. T. Grafton, and M. B. Miller, "Individual differences in shifting decision criterion: A recognition memory study," *Mem. Cogn.*, vol. 40, pp. 127–134, 2012.

[4] M. I. Posner, "Orienting of attention," *Quarterly Journal of Experimental Psychology*, vol. 32, pp. 3–25, 1980.

[5] D. S. Bassett, J. A. Brown, V. Deshpande, J. M. Carlson, and S. T. Grafton, "Conserved and variable architecture of human white matter connectivity," *NeuroImage*, vol. 54, pp. 1262–1279, 2011.

[6] V. J. Wedeen, D. L. Rosene, R. Wang, G. Dai, F. Mortazavi, P. Hagmann, and J. H. K. ansW Y I. Tseng, "The geometric structure of the brain fiber pathways," *Science*, vol. 335, pp. 1628–1634, 2012.

[7] G. J. Parker and D. C. Alexander, "Probabilistic Monte Carlo based mapping of cerebral connections utilising whole-brain crossing fibre information," *Information Processing in Medical Imaging*, vol. 18, pp. 684–695, 2003.

[8] A. Krishnan, L. J. Williams, A. R. McIntosh, and H. Abdi, "Partial least squares (pls) methods for neuroimaging: a tutorial and review.," *NeuroImage*, vol. 56, no. 2, pp. 455–475, 2011.

[9] J. X. O'Reilly, M. W. Woolrich, T. E. Behrens, S. M. Smith, and H. Johansen-Berg, "Tools of the trade: psychophysiological interactions and functional connectivity," *Soc. Cogn. Affect. Neurosci.*, vol. 7, no. 5, pp. 604–609, 2012.

[10] D. S. Bassett, B. G. Nelson, B. A. Mueller, J. Camchong, and K. O. Lim, "Altered resting state complexity in schizophrenia," *NeuroImage*, vol. 59, no. 3, pp. 2196–2207, 2012.

[11] A. Zalesky, A. Fornito, and E. Bullmore, "On the use of correlation as a measure of network connectivity," *NeuroImage*, vol. 60, no. 4, pp. 2096–2106, 2012.

[12] N. Petridou, C. C. Gaudes, I. L. Dryden, S. T. Francis, and P. A. Gowland, "Periods of rest in fmri contain individual spontaneous events which are related to slowly fluctuating spontaneous activity," *Hum. Brain Mapp.*, 2012.

[13] Tzourio-Mazoyer, B. Landeau, D. Papathanassiou, F. Crivello, O. Etard, N. Delcroix, B. Mazoyer, and M. Joliot, "Automated anatomical labeling of activations in spm using a macroscopic anatomical parcellation of the mni mri single-subject brain," *NeuroImage*, vol. 15, pp. 273–289, 2002.

[14] M. A. Porter, J.-P. Onnela, and P. J. Mucha, "Communities in networks," *Not Amer Math Soc*, vol. 56, no. 9, pp. 1082–1097, 1164–1166, 2009.

[15] S. Fortunato, "Community detection in graphs," *Phys Rep*, vol. 486, no. 3–5, pp. 75–174, 2010.

[16] D. S. Bassett, M. A. Porter, N. F. Wymbs, S. T. Grafton, J. M. Carlson, and P. J. Mucha, "Robust detection of dynamic community structure in networks," *Chaos*, vol. 23, no. 1, p. 013142, 2012.

[17] M. E. J. Newman and M. Girvan, "Finding and evaluating community structure in networks," *Phys Rev E*, vol. 69, p. 026113, 2004.

[18] M. E. J. Newman, "Fast algorithm for detecting community structure in networks," *Phys Rev E*, vol. 69, p. 066133, 2004.

[19] M. E. J. Newman, "Modularity and community structure in networks," *Proc Natl Acad Sci USA*, vol. 103, pp. 8577–8582, 2006.

[20] J. Reichardt and S. Bornholdt, "Statistical mechanics of community detection," *Phys Rev E*, vol. 74, p. 016110, 2006.

[21] J.-P. Onnela, D. J. Fenn, S. Reid, M. A. Porter, P. J. Mucha, M. D. Fricker, and N. S. Jones, "Taxonomies of networks," *Phys Rev E, in press (arXiv:1006.5731)* , 2012.

[22] U. Brandes, D. Delling, M. Gaertler, R. Görke, M. Hoefer, Z. Nikoloski, and D. Wagner, "On modularity clustering," *IEEE Trans on Knowl Data Eng*, vol. 20, pp. 172–188, 2008.

[23] A. L. Traud, E. D. Kelsic, P. J. Mucha, and M. A. Porter, "Comparing community structure to characteristics in online collegiate social networks," *SIAM Rev*, vol. 53, pp. 526–543, 2011.
